# Combining Ordinary Kriging with wind directions to identify sources of industrial odors in Portland, Oregon

**DOI:** 10.1371/journal.pone.0189175

**Published:** 2018-01-31

**Authors:** Ted C. Eckmann, Samantha G. Wright, Logan K. Simpson, Joe L. Walker, Steven A. Kolmes, James E. Houck, Sandra C. Velasquez

**Affiliations:** Department of Environmental Studies, University of Portland, Portland, Oregon, United States of America; Coastal Carolina University, UNITED STATES

## Abstract

This study combines Ordinary Kriging, odor monitoring, and wind direction data to demonstrate how these elements can be applied to identify the source of an industrial odor. The specific case study used as an example of how to address this issue was the University Park neighborhood of Portland, Oregon (USA) where residents frequently complain about industrial odors, and suspect the main source to be a nearby Daimler Trucks North America LLC manufacturing plant. We collected 19,665 odor observations plus 105,120 wind measurements, using an automated weather station to measure winds in the area at five-minute intervals, logging continuously from December 2014 through November 2015, while we also measured odors at 19 locations, three times per day, using methods from the American Society of the International Association for Testing and Materials. Our results quantify how winds vary with season and time of day when industrial odors were observed versus when they were not observed, while also mapping spatiotemporal patterns in these odors using Ordinary Kriging. Our analyses show that industrial odors were detected most frequently to the northwest of the Daimler plant, mostly when winds blew from the southeast, suggesting Daimler’s facility is a likely source for much of this odor.

## Introduction

This study combines Ordinary Kriging, odor monitoring, and wind direction data to demonstrate how these elements can be combined to identify the source of an industrial odor. The specific case study used as an example of how to address this issue was the University Park neighborhood of Portland, Oregon (USA) where residents frequently complain about industrial odors, and suspect the main source to be a nearby Daimler Trucks North America LLC manufacturing plant. Daimler manufactures and paints heavy trucks at this facility, and therefore we also examined qualitative data on odor type to assess how common paint odors were near the Daimler facility as compared to the rest of the study area, and to aid in determining if the industrial odors could be from other potential sources, such as a bulk gasoline terminal to the west of the study area, or a shipyard to the south of the study area.

**Fig 1:**
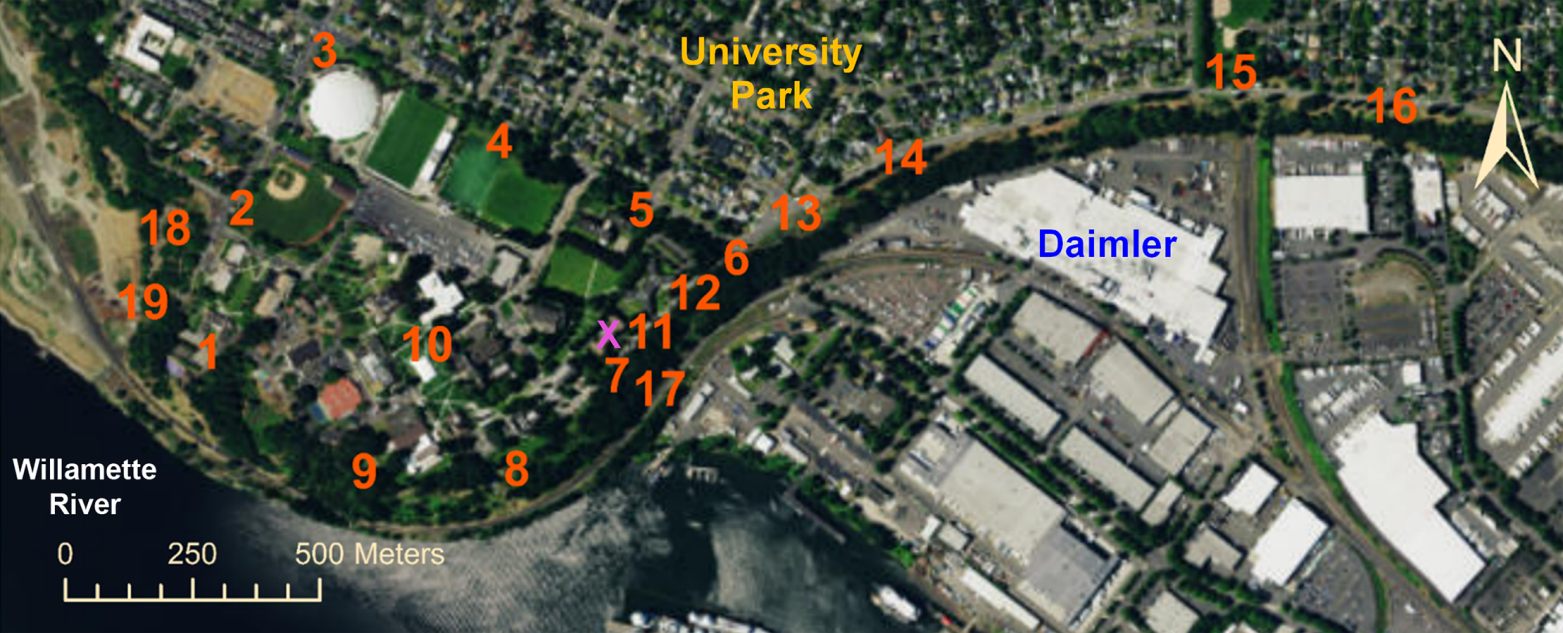
The study area in Portland, Oregon (USA). Orange numbers indicate locations of the 19 stations where this study collected odor data three times per day for one year. The blue label “Daimler” represents the location of the Daimler Trucks North America LLC plant this study hypothesized as producing industrial odors in the area. The yellow label “University Park” represents the location of a residential area on a bluff ~40 meters above the Daimler plant. The University of Portland campus is southwest of the University Park neighborhood. Odor detection stations were located subject to limited resources such as labor, and obstacles such as steep terrain and private property: all stations are either on public and easily accessible property, or on the University of Portland campus. The pink “X” shows the location of the automated weather station this study installed to measure wind speeds and directions in the area. Basemap from Esri’s ArcGIS.

We collected 19,665 odor observations plus 105,120 wind measurements, using an automated weather station to measure winds at five-minute intervals, logging continuously from December 2014 through November 2015, while we also measured odors at 19 locations, three times per day, using methods from the American Society of the International Association for Testing and Materials (ASTM). Our results quantify how winds vary with season and time of day when industrial odors were observed versus when they were not observed, while also mapping spatiotemporal patterns in these odors using Ordinary Kriging. The Northwest Region of the Oregon Department of Environmental Quality (hereinafter DEQ), using limited resources, conducted a study from October 2014 through October 2015 which concluded that Daimler was not a source of nuisance odors [1]. The goals of our larger parallel study were to 1) map the distribution of industrial odors in University Park, 2) examine winds during odor events, 3) assess how both of these vary by season and time of day, 4) identify sources of the odors, and 5) suggest potential solutions to this problem. Our study, based upon the spatial relationship of public odor complaints to the Daimler facility, the nature and locations of other industrial facilities in that area, and following the design of DEQ’s study, hypothesized the main source of industrial odors in this area to be the Daimler facility. To test this hypothesis, we set up 19 stations in the area to monitor odors three times per day for a full year, plus one automated weather station to measure winds (Fig 1).

Commonly, nuisance industrial odors are produced by volatile organic compounds (VOC) at low and ephemeral atmospheric concentrations. Low concentrations and short-term impacts coupled with variable individual olfactory thresholds, variable hedonic perception, and olfactory fatigue caused by prolonged or repetitive exposure, make nuisance odors difficult to quantify. Significant temporal changes in meteorological conditions further complicate the issue in this case: the study area of Portland, Oregon exhibits significant variability in winds [2] and other atmospheric parameters [3] on seasonal and diurnal timescales. It has been found that in some cases, fewer than 15% of residents in a community impacted by nuisance odors file complaints [4]. This is unfortunate because odors can cause symptoms beyond simple annoyance, as studies have found they are often related to a variety of health problems [5].

The DEQ study assessed odors by sending several staff members to locations in and near the area shown in Fig 1, after giving these staff members an odor sensitivity screening that found they had odor sensitivity within an acceptable range for humans, and evaluated for odor intensity using an n-butanol standard [1]. DEQ staff evaluated odors at eight to ten locations within and near the study area, on 80 different dates, averaging two to three times per week. DEQ staff did not detect any odors at all in the last five months of their investigation, and stated that they found “no observable odor patterns or correlations to the time of day” [1]. DEQ staff also spent a minimum duration of five minutes at each location, and if they observed an odor during the first five minutes, remained in that location until they no longer detected the odor. However, this does not necessarily measure the duration of the odor [6], because the widely-documented process of olfactory fatigue [7] can reduce human sensitivity to an odor after exposures for as short as just a few minutes [8].

To both incorporate established odor survey techniques and align our odor study as much as possible with the DEQ regulatory process, we followed similar procedures, such as using human odor monitors trained and tested by use of an n-butanol standard, but we observed much more frequently and consistently, making the same number of observations in each season and time of day. We also used more stations and visited each station the same number of times, remaining at each station for the same amount of time, in order to avoid spatial or temporal bias in data collection. We followed DEQ in using field surveys with trained human assessors because many studies, such as those described in the 2013 review article by Capelli *et al*. [9], found these methods to be superior to other approaches such as mathematical modeling of emission plumes and their dispersion, physical and chemical measurements, and a class of fast gas chromatographs, mass spectrometers, and other gas sensors known as ‘electronic noses.’ In describing drawbacks of dispersion models for applications such as ours where information about the source is limited, this recent review article states “fugitive sources are hardly modelled, because of uncertainties regarding timing, location and emission rates” [9]. The article also states that chemical analysis, in situations like ours where the source could include multiple compounds, is “unsuitable for the purpose of determining the presence of odours in the environment” [9], while also describing limitations of electronic noses, as more recent studies have also done [10]. While finding that the main disadvantage of trained human odor observers is the large labor cost involved relative to these other methods, especially dispersion models, the review article states in its highlights section that humans “are necessary for direct assessment of odour in the field,” hence our use of humans for our field odor study [9]. Another disadvantage of human odor observers relative to dispersion models is that models can be used for predictive [9] and hypothetical scenarios, though these are not relevant to our application, so we focused on thorough data gathering and analysis using human odor observation rather than splitting our resources between those techniques and dispersion modeling.

Our human odor observers were first trained and tested for their olfactory sensitivity by the use of the ASTM E544-10 standardized olfactory perception practice for determining odor type and concentration [11]. The choice of ASTM E544-10 is supported quantitatively by a careful comparison of seven different well-established techniques for odor detection that showed, using least significant difference (LSD) multiple comparison results, that all of the common field techniques tested were statistically indistinguishable from one another in their session means, and only a laboratory-based dynamic triangular forced‐choice olfactometry (DTFCO) approach (that collected samples in Tedlar bags for later laboratory evaluation by a group of panelists) produced significantly different results, with the latter difference probably due to the collection bags having had a detectable background odor [12]. The ASTM E544-10 is an American standard closely related to the German VDI 3940 [13] technique, and is used in our study in a grid technique [14] similar to that of VDI 3940 [15] with sampling locations appropriate to the unusual topography of our study’s location. Our odor observers were first trained in odor detection, as described in the Methods section below, and our sampling sites along the top of a ~40 m tall bluff were organized to avoid the “vicinity of houses, high walls, fences, edge of forest, roads with heavy traffic, railways, bus stops and taxi ranks” as recommended by Sówka [16]. Our study relied on a regular set of observations rather than being carried out with *ad hoc* observation locations in a shifting plume as a VDI 3940 plume technique would be [16]. Odor measurements following VDI 3940 methods have also been statistically analyzed with Kriging [17] to assess the spatial extent of odor nuisance [18], and our study employs Kriging for statistical analysis as well, as described in detail by our Methods section.

Our study provided the opportunity to attempt to disprove the hypothesis that Daimler is the main source of industrial odors detected in the nearby residential neighborhood by collecting data on odor type at 19 locations three times per day for a year, measuring winds throughout the year, and analyzing patterns in odors through Kriging along with their relationships to winds. Three results would allow that hypothesis to be considered disproven: (1) Kriging might indicate industrial odors are not statistically more likely to be present near Daimler than in other parts of the study area, (2) the type of odors more frequently observed near Daimler may be inconsistent with Daimler being their source (*e*.*g*. are they paint odors, or from a process not present at Daimler?), or (3) winds might not be consistent with odors being carried from the Daimler facility to the residential neighborhood.

## Methods

All meteorological data for our study were collected through an automated weather station using an Onset HOBO U30 data logger (Onset Corp., Bourne, MA), which we installed on the roof of a three-story dormitory building at the University of Portland campus in Portland, Oregon (USA). This building and the University Park neighborhood are situated on a bluff ~40 meters above the hypothesized main source of the pollutants (Fig 1). Table 1 shows relevant specifications for this weather station, which included a wind vane (S-WDA-M003) for wind direction measurements, and an independent anemometer (S-WSA-M003) for wind speed measurements, which this study purchased as a joint product called the Wind Smart Sensor Set (S-WSET-A, Onset Corp., Bourne, MA). Other studies have used these sensors before, both in air pollution research [19], and in other applications [20]. We placed both wind sensors three meters above rooftop level, and the data logger recorded a new data point from each wind sensor every five minutes as an average for the preceding five minutes. This data logger collected measurements continuously from the beginning of our study period (December 1, 2014) through the end of our study period (November 30, 2015), with no data gaps (S1 Dataset).

**Table 1:** Manufacturer specifications for the sensors on the automated weather station this study deployed.

	Wind Speed (S-WSA-M003)	Wind Direction (S-WDA-M003)
**Accuracy**	± 1.1 m/s or ± 4% of reading, whichever is greater	± 5 degrees
**Resolution**	0.5 m s^-1^	1.4 degrees
**Starting Threshold**	≤1.0 m s^-1^	1.0 m s^-1^

For reasons described above in the Introduction section, and in order to make the results of this study as applicable as possible to Oregon State law regarding nuisance odors [1], this study assessed odors as detected by humans trained in the ASTM E544-10 standardized olfactory perception practice for determining odor type and concentration [11]. This ASTM method involves using various concentrations of known chemicals to train people in odor detection and to test them for their ability to detect an odor and discern its type. Human monitors for this study had to pass an odor intensity scale exam, which involved correctly sorting ten unlabeled flasks with different ratios of water and a reference odorant, 1-butanol (n-butanol), from lowest concentration to highest. The ASTM E544-10 method, using a geometric progression scale with a ratio of two, is implemented by comparing the odor intensity of each sample to the odor intensities of a concentration series of a reference chemical. A set of Erlenmeyer flasks, containing different concentrations of n-butanol in water, was used for training our odor observers. Employing these methods assured that all of our observers were able to detect subtle odor distinctions well, and were more than able to collect the binary data (see below) used in our analysis.

Once trained and tested, observers recorded data in a binary “present/absent” form for analysis that maximized inter-observer reliability. The use of ASTM E544-10 for our training constituted use of one of the most reliable recognized techniques, as indicated by Brancher *et al*. [21]: “Odour intensity is quantified based on reference scales, where the perceived intensity of an odour is compared to the intensity of a standard chemical substance (n-butanol for olfactometry). The main reference scales standards for odour intensity measurement are from Germany: VDI 3882—Part 1:1992 (VDI, 1992) [22]; U.S.: ASTM E544-10 (ASTM, 2010) [11]; France: AFNOR X 43–103 (AFNOR, 1993) [23].” In terms of comparisons to our research, as noted by Brancher *et al*. [21] the use of VDI 3940 rather than VDI 3882 is appropriate for grid or plume measurements, as in the case of the grid measurements made in this study. The human odor detectors for our study were trained and tested by Environmental Resources Management (ERM, http://www.erm.com/) in Portland, OR. At the time of this study, all of these human odor monitors were undergraduate students attending the University of Portland, and of similar ages, which is important because studies have found that olfactory sensitivity generally decreases with age [24]. Our study coordinated odor monitors for consistent classification of all odors as either “industrial” or “non-industrial.” Human odor monitors were also asked to provide descriptive terms for all industrial odors, and the most common subcategories they provided for industrial odors were: “paint,” “chemical,” and “petroleum.” Likewise, these human monitors divided non-industrial odors into the following subcategories: “vegetative,” “wood smoke,” “vehicular exhaust,” and “food preparation.”

This study only presents full analyses for the “industrial” category to focus on the problem reported by residents in the area, though the results section also presents comparisons of winds measured when food preparation odors were detected, as compared to winds measured when industrial odors were detected, in order to assess the reliability of the qualitative human categorization of these odors. We selected the food preparation subcategory to assess the reliability of odor categorization because the houses in the University Park neighborhood are easily identifiable and stationary sources of food preparation odors, but are located in a different part of the study area from the Daimler plant (Fig 1), and the winds should thus be different when industrial odors were detected as compared to when food preparation odors were detected. Our study’s wind data were not made available to any of the human detectors until after the completion of all odor observations, which reduced the likelihood of any possible intentional or unintentional bias on the part of the human detectors: as the results section later shows, the wind data, which were measured independently by an automated device, corroborate the patterns in the human-measured odor data. Odor monitors measured at 19 predetermined locations (Fig 1), hereafter referred to as odor detection stations, which were spatially distributed in order to 1) detect odors coming from any direction, 2) assess if industries other than Daimler might also be producing some of the odors that have drawn complaints from residents in the study area, and 3) to compare major odor sources with background odor levels in the area. Odor data collection occurred at each of these 19 stations three times per day: 06:00 to 08:00 local time (hereinafter “morning”), 11:00 to 13:00 local time (hereinafter “midday”), and 16:00 to 18:00 local time (hereinafter “evening”), from the beginning of the study period (December 1, 2014) through the end of the study period (November 30, 2015). Unlike the automated weather station, odor data for some days are not available, mostly due to scheduling problems for the students: we only collected odor observations on 95% of the days during the study period. On all days with available data, odor data sets were complete as we made observations at all 19 stations during all three of the designated times of day.

Industrial odors are less likely to be present on the days when we did not make observations because some of them were holidays when most local industries were not operating, such as Christmas Day. Thus, the effect of these missing days likely biases our results towards slightly higher overall frequencies of industrial odors than are actually present, but this effect is likely to be small considering only 5% of days during the study period went un-sampled, and fewer than 5% of these were days when most local industries were not operating. As the results section later shows, this potential source of bias is very small compared to the patterns we observed, and thus it is very unlikely to affect the reliability of our conclusions (S2 Dataset).

In all cases, the same human odor monitor visited all 19 stations exactly once for each observation period, in order not to bias observations by having one person visit some stations and a different person visit the others. Each odor monitor spent the same amount of time at each station on each monitoring day (in each case under one minute to provide a relatively instantaneous snapshot with a binary yes/no for odor) so that the total amount of time spent monitoring at each station is the same.

All 19 stations were located either on public property or on the University of Portland campus and accessed with permission from the University of Portland. No permits were required for the described study, which complied with all relevant regulations. Limitations on available resources such as labor, plus obstacles in the way of sampling, such as very steep terrain and private properties owned by other parties, affected where we could position these odor detection stations, and the number of stations we could employ. We established the stations at locations spaced out largely along the top of a ~40 m bluff, at locations between the street at the top and the edge of the bluff where there were no barriers to access due to large bushes and/or trees, no guard rails or metal fences delimiting unstable slopes, flat ground with sufficient width for the odor observers and their bicycles to stand entirely off the public street and its traffic, no bus stops to interfere with, and not immediately adjacent to any houses, following practices recommended by other studies [16]. The resulting spatial distribution of stations provided many opportunities to reject the hypothesis that Daimler was the main source of industrial odors in the area, because many of our stations are well west of Daimler, towards a bulk gasoline facility, and south of Daimler, towards a shipyard (Fig 1). However, the resource and siting considerations described above required that many locations within the study area did not have a station. Therefore, to analyze the geographic patterns of odors in the study area at all locations, including those without odor stations, and to provide robust statistical assessments of results, this study employed a spatial interpolation method called Kriging [25].

Much like a widely-used spatial interpolation approach called inverse distance weighting [26], Kriging estimates values at locations between measurement sites by weighting values measured nearby more strongly than it weights values measured at greater distances. Kriging also uses geostatistical models that consider spatial autocorrelation, unlike the comparatively simpler methods of inverse distance weighting, and thus Kriging produces better results in many situations [27]. Kriging is also effective even with very few input locations [28], making it particularly well-suited to our application.

This study mapped all station data and performed Kriging calculations using Esri’s ArcGIS. We employed a type of Kriging called Ordinary Kriging because we had no reason to doubt that odors would exhibit an approximately normal distribution [29], while one of the main disadvantages of Ordinary Kriging (producing unrealistically smooth outputs [30]) is not detrimental to the nature of the data in our application (odors) [18] or our goals of identifying their sources in combination with wind data. This study utilized a Gaussian model [31] with a variable search radius because those settings produced the best fit for spatial patterns in the data, and this model is appropriate because it does not assume that the odor was produced at each station. We used the ArcGIS defaults for all other settings and parameters. A corresponding standard error map [32] was created to correspond to each odor map in order to describe the uncertainty in modeled frequency of detected odors in the areas surrounding each station.

## Results

Table 2 displays the total number of detections by odor type, and also compares the total detected by our study at all 19 stations to just those detections from stations 13 and 14. Table 2 shows that 49% of all paint detections occurred at stations 13 and 14, which are the two stations closest to Daimler’s facility that manufactures and paints heavy trucks [1]. This 49% is substantially higher than the overall average for the neighborhood, both for the “paint” odor subcategory, and for the broader “industrial” odor category. From 19,665 odor observations made at our 19 stations, both of these results would be very unlikely to occur if the source of much of these odors was not closer to stations 13 and 14 than to any of our other stations.

**Table 2:** Number of odor detections by type and location. This shows the total number of odor detections this study recorded from December 2014 through November 2015, by type of odor, separating the industrial and non-industrial categories into subcategories. The table also compares the total from all 19 stations to just those from stations 13 and 14 (see map in Fig 1).

Odor Type	Total From All 19 Stations	From Only Stations 13 and 14
**Industrial**		
**Paint**	190	93
**Other Industrial**	380	30
**Non-Industrial**		
**Vehicle Exhaust**	1468	231
**Wood Smoke**	218	39
**Food Preparation**	347	16
**Vegetative**	3622	433
**Total Odor Detections**	6225	842

**Fig 2:**
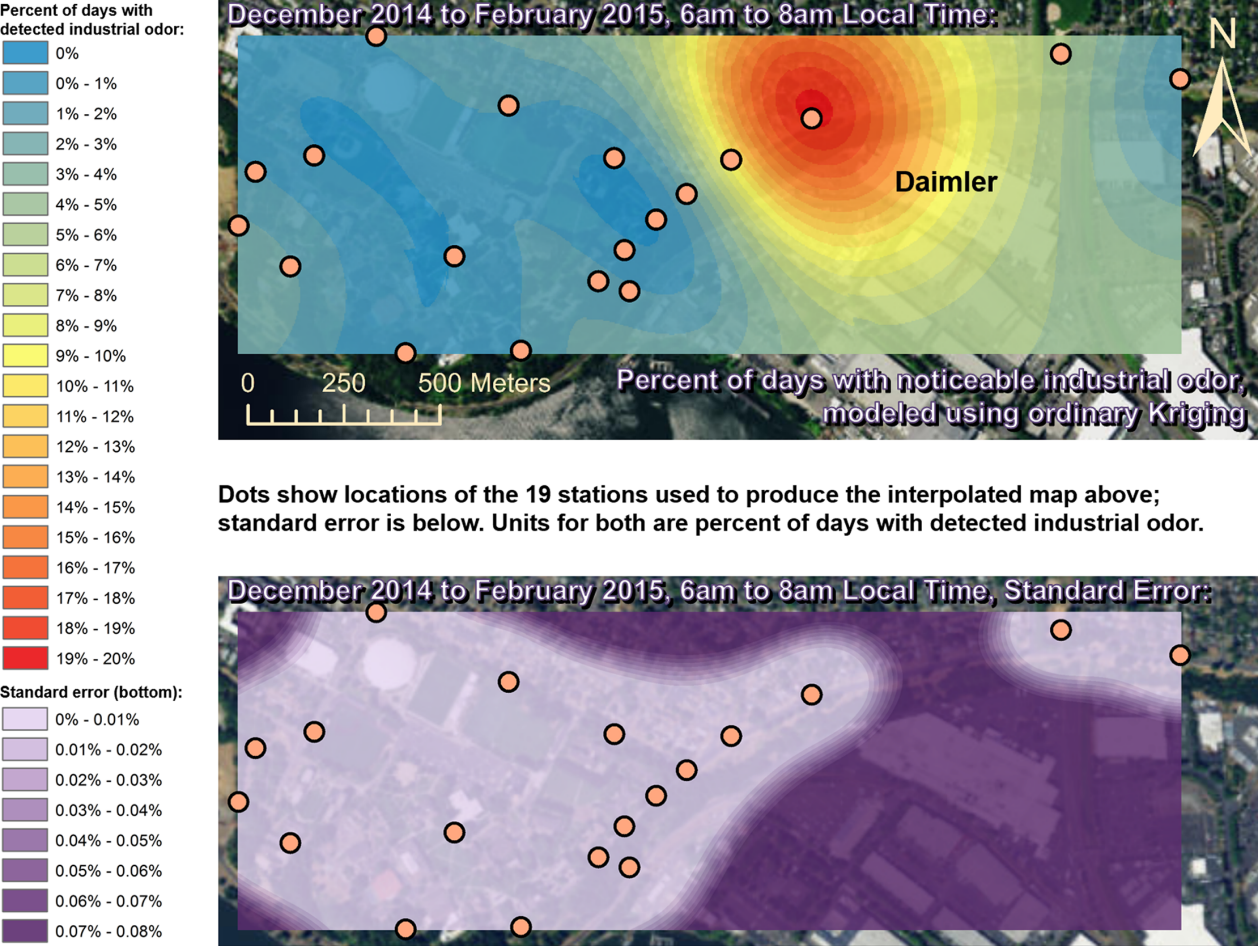
Frequencies of industrial odor detections (above) and standard error (below) during morning in winter. The areas northwest of the Daimler plant experienced far more industrial odors during this period than any other areas in the map according to both raw station data and the spatial interpolation here (above). These frequencies of odor detections are all much larger than the standard errors from the interpolation shown here (below) indicating a high signal-to-noise ratio. As expected, standard errors generally increase with increasing distance from the stations used to produce the interpolation (the dots shown here). The bins here include all the values (no locations exceeded 20% of days with detected odors, or a standard error of 0.08%). Basemap from Esri’s ArcGIS.

**Fig 3:**
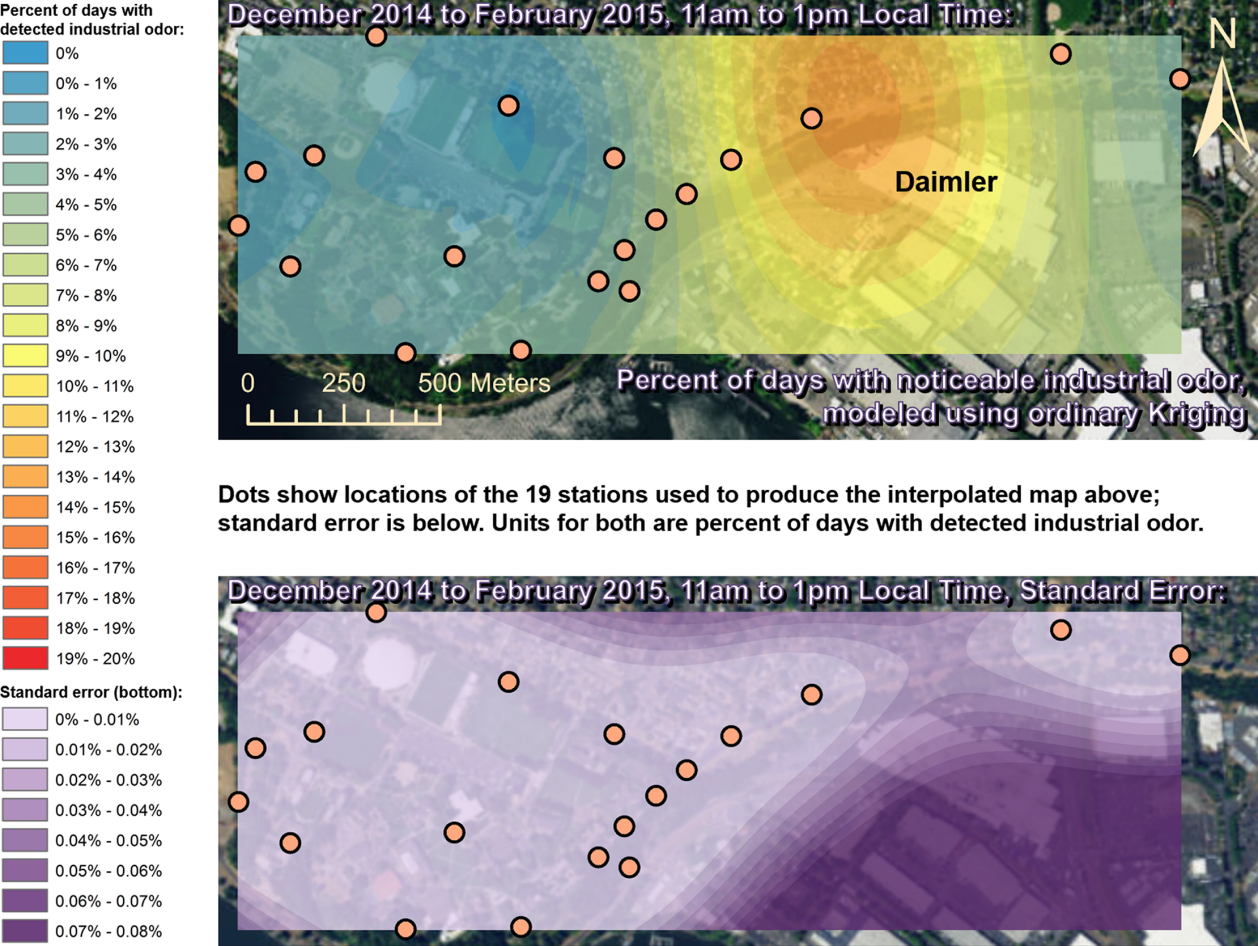
Frequencies of industrial odor detections (above) and standard error (below) during midday in winter. The areas northwest of the Daimler plant experienced far more industrial odors during this period than any other areas in the map according to both raw station data and the spatial interpolation here (above). These frequencies of odor detections are all much larger than the standard errors from the interpolation shown here (below) indicating a high signal-to-noise ratio. As expected, standard errors generally increase with increasing distance from the stations used to produce the interpolation (the dots shown here). The bins here include all the values (no locations exceeded 20% of days with detected odors, or a standard error of 0.08%). Basemap from Esri’s ArcGIS.

**Fig 4:**
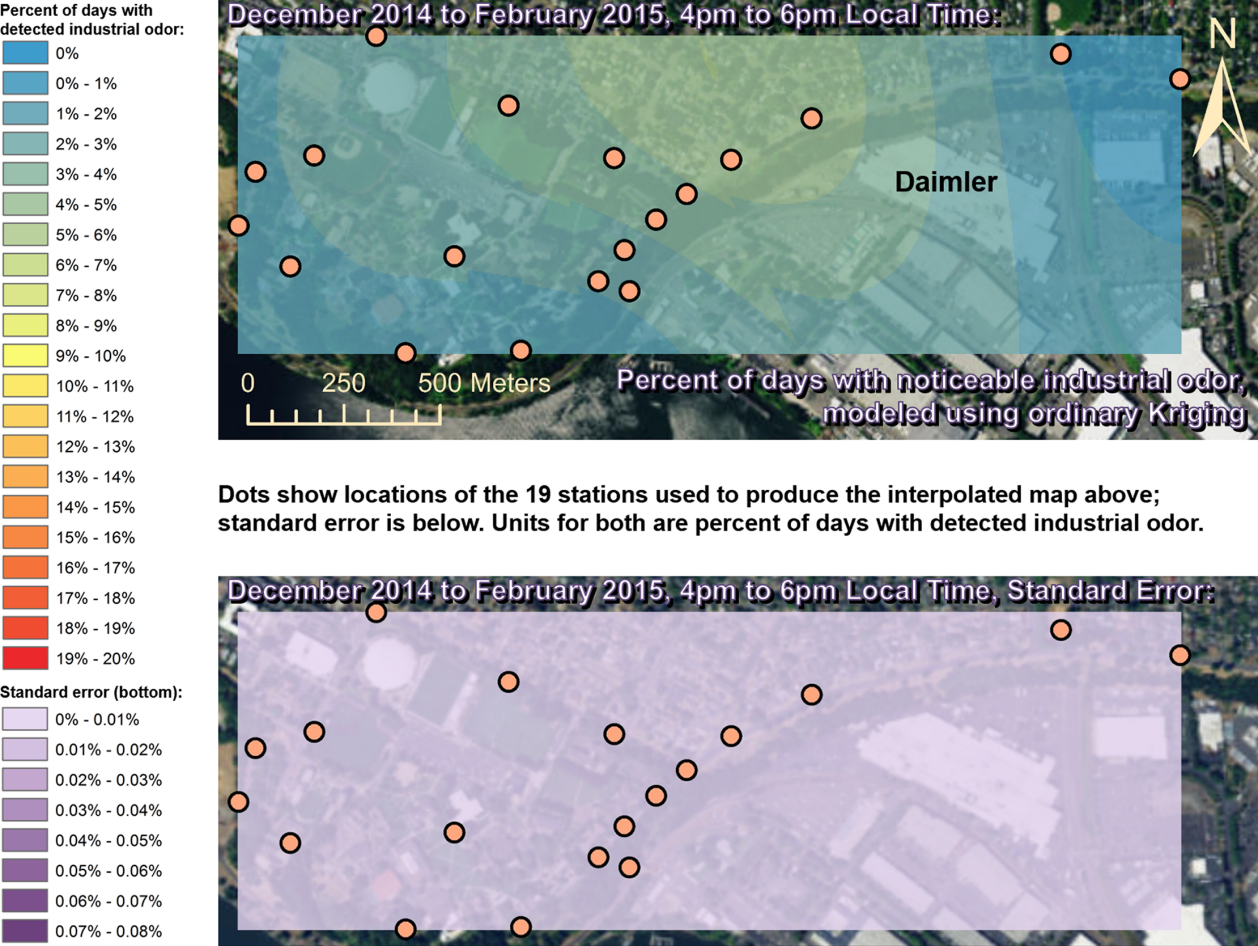
Frequencies of industrial odor detections (above) and standard error (below) during evening in winter. The areas northwest of the Daimler plant experienced slightly more industrial odors during this period than any other areas in the map according to both raw station data and the spatial interpolation here (above). These frequencies of odor detections are all much larger than the standard errors from the interpolation shown here (below) indicating a high signal-to-noise ratio. As expected, standard errors generally increase with increasing distance from the stations used to produce the interpolation (the dots shown here). The bins here include all the values (no locations exceeded 20% of days with detected odors, or a standard error of 0.08%). Basemap from Esri’s ArcGIS.

**Fig 5:**
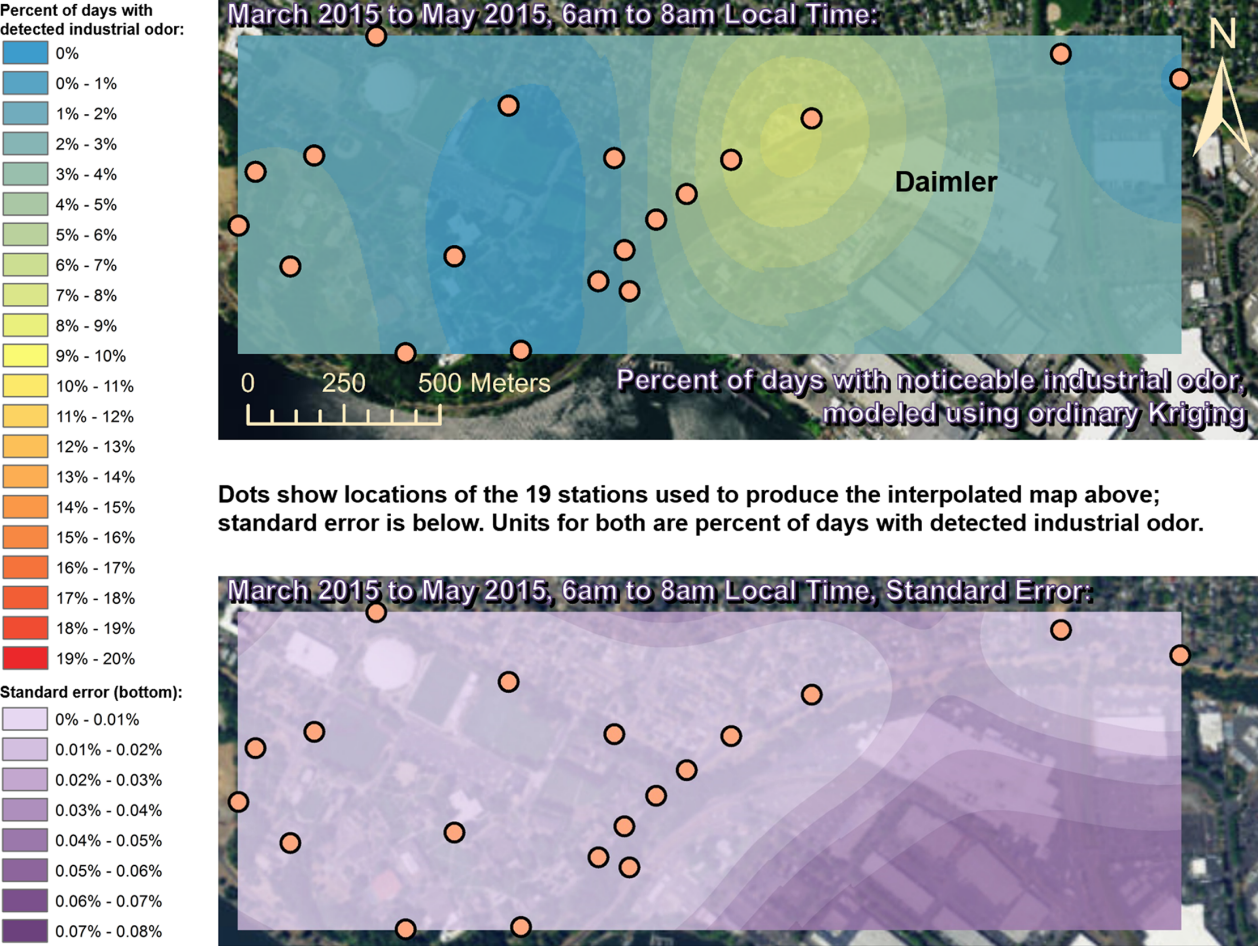
Frequencies of industrial odor detections (above) and standard error (below) during morning in spring. The areas northwest of the Daimler plant experienced far more industrial odors during this period than any other areas in the map according to both raw station data and the spatial interpolation here (above). These frequencies of odor detections are all much larger than the standard errors from the interpolation shown here (below) indicating a high signal-to-noise ratio. As expected, standard errors generally increase with increasing distance from the stations used to produce the interpolation (the dots shown here). The bins here include all the values (no locations exceeded 20% of days with detected odors, or a standard error of 0.08%). Basemap from Esri’s ArcGIS.

**Fig 6:**
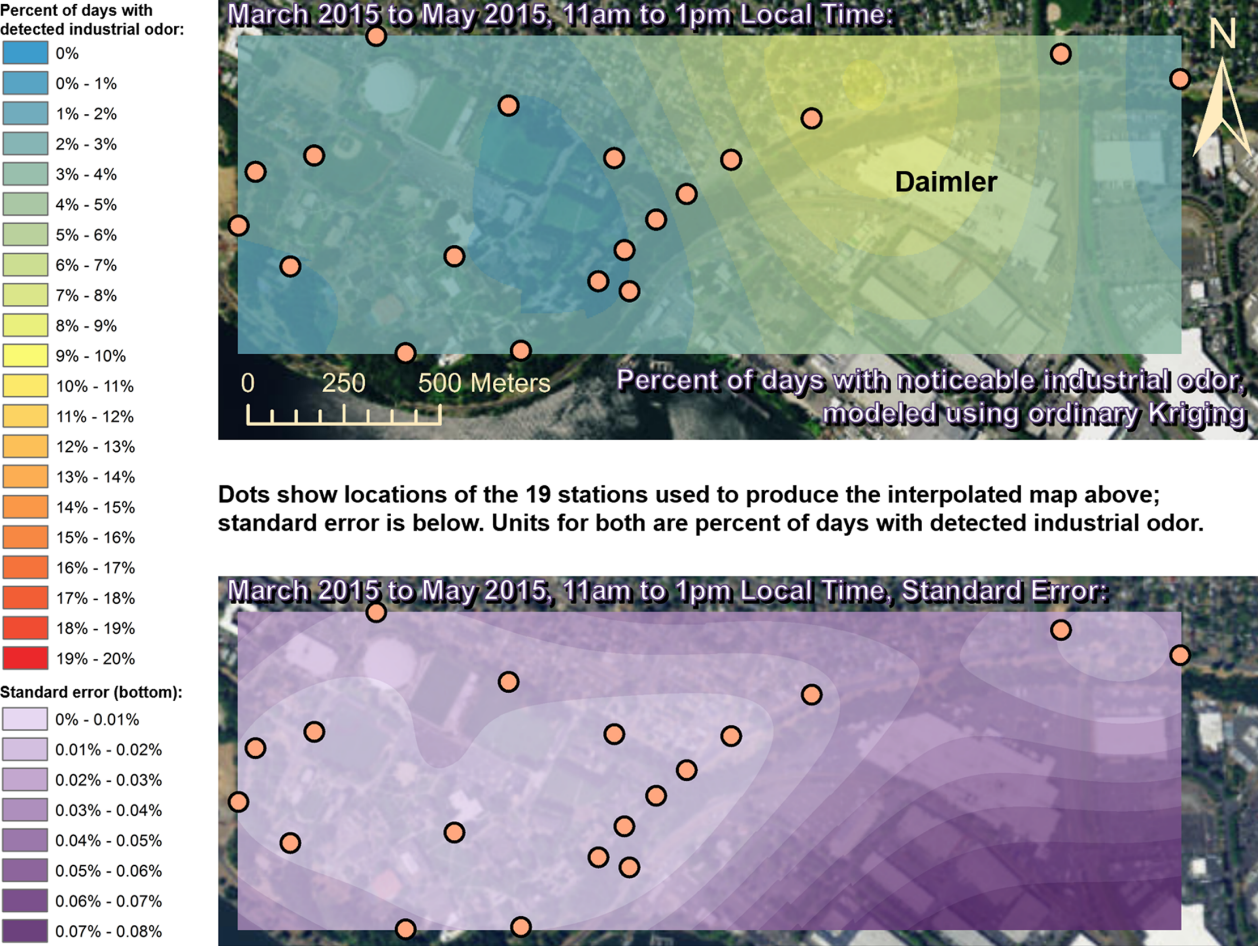
Frequencies of industrial odor detections (above) and standard error (below) during midday in spring. The areas northwest of the Daimler plant experienced far more industrial odors during this period than any other areas in the map according to both raw station data and the spatial interpolation here (above). These frequencies of odor detections are all much larger than the standard errors from the interpolation shown here (below) indicating a high signal-to-noise ratio. As expected, standard errors generally increase with increasing distance from the stations used to produce the interpolation (the dots shown here). The bins here include all the values (no locations exceeded 20% of days with detected odors, or a standard error of 0.08%). Basemap from Esri’s ArcGIS.

**Fig 7:**
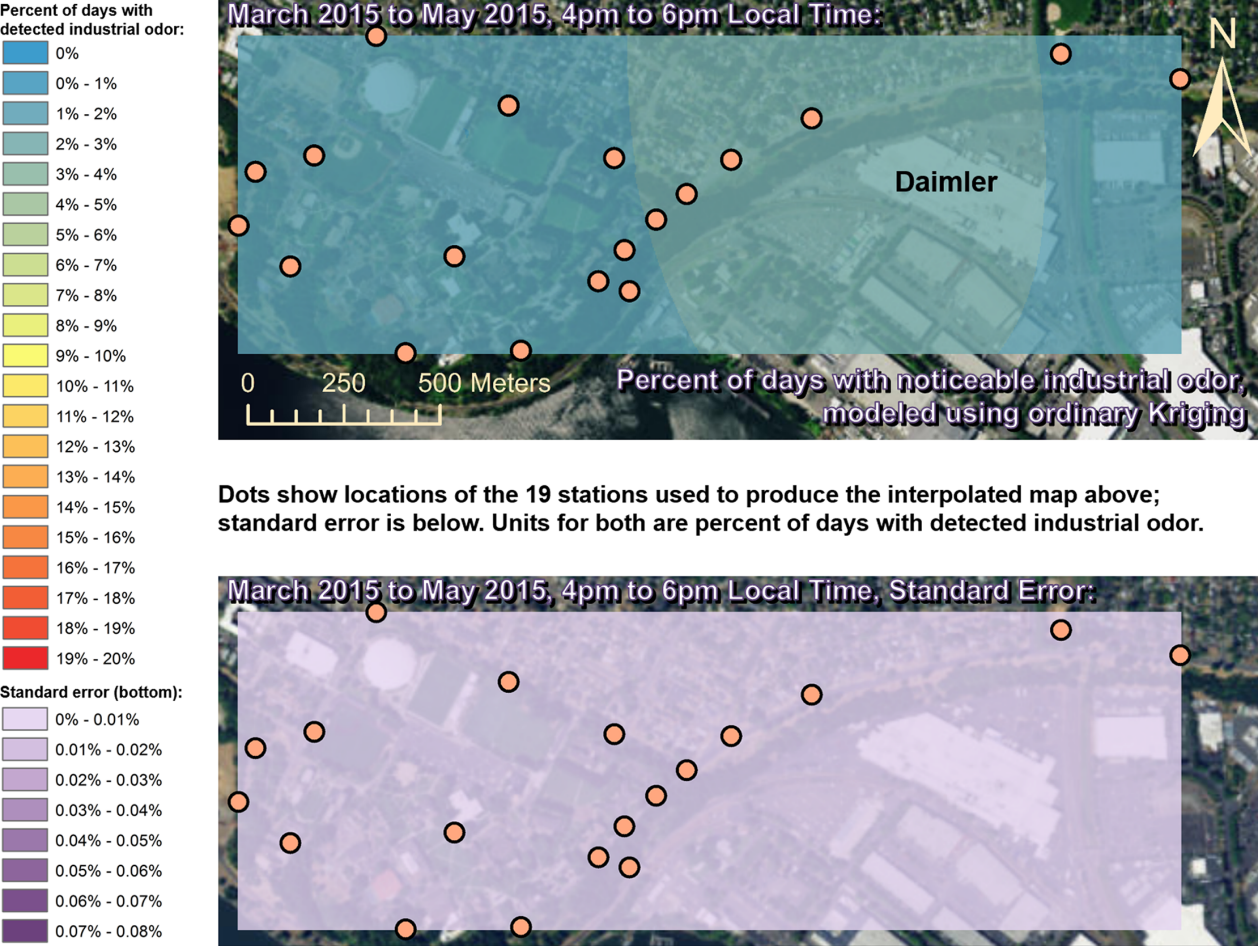
Frequencies of industrial odor detections (above) and standard error (below) during evening in spring. The areas surrounding the Daimler plant experienced slightly more industrial odors during this period than any other areas in the map according to both raw station data and the spatial interpolation here (above). These frequencies of odor detections are all much larger than the standard errors from the interpolation shown here (below) indicating a high signal-to-noise ratio. As expected, standard errors generally increase with increasing distance from the stations used to produce the interpolation (the dots shown here). The bins here include all the values (no locations exceeded 20% of days with detected odors, or a standard error of 0.08%). Basemap from Esri’s ArcGIS.

**Fig 8:**
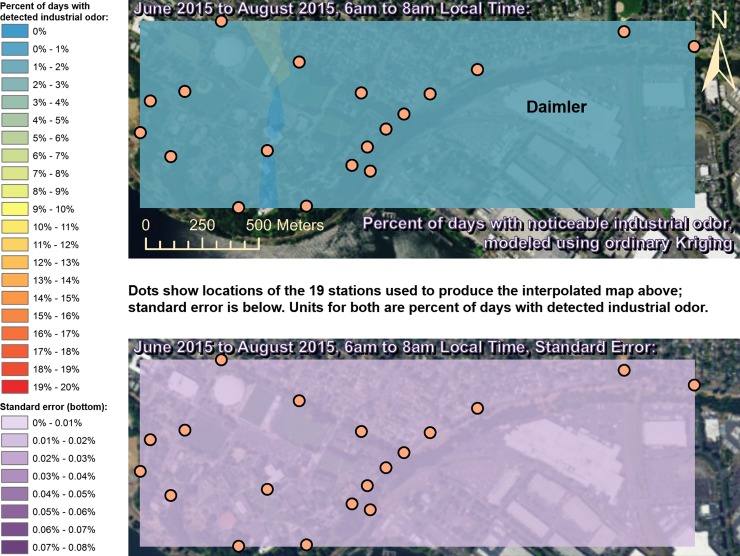
Frequencies of industrial odor detections (above) and standard error (below) during morning in summer. The spatial distribution of odor detections shows no clear pattern during this period. The bins here include all the values (no locations exceeded 20% of days with detected odors, or a standard error of 0.08%). Basemap from Esri’s ArcGIS.

**Fig 9:**
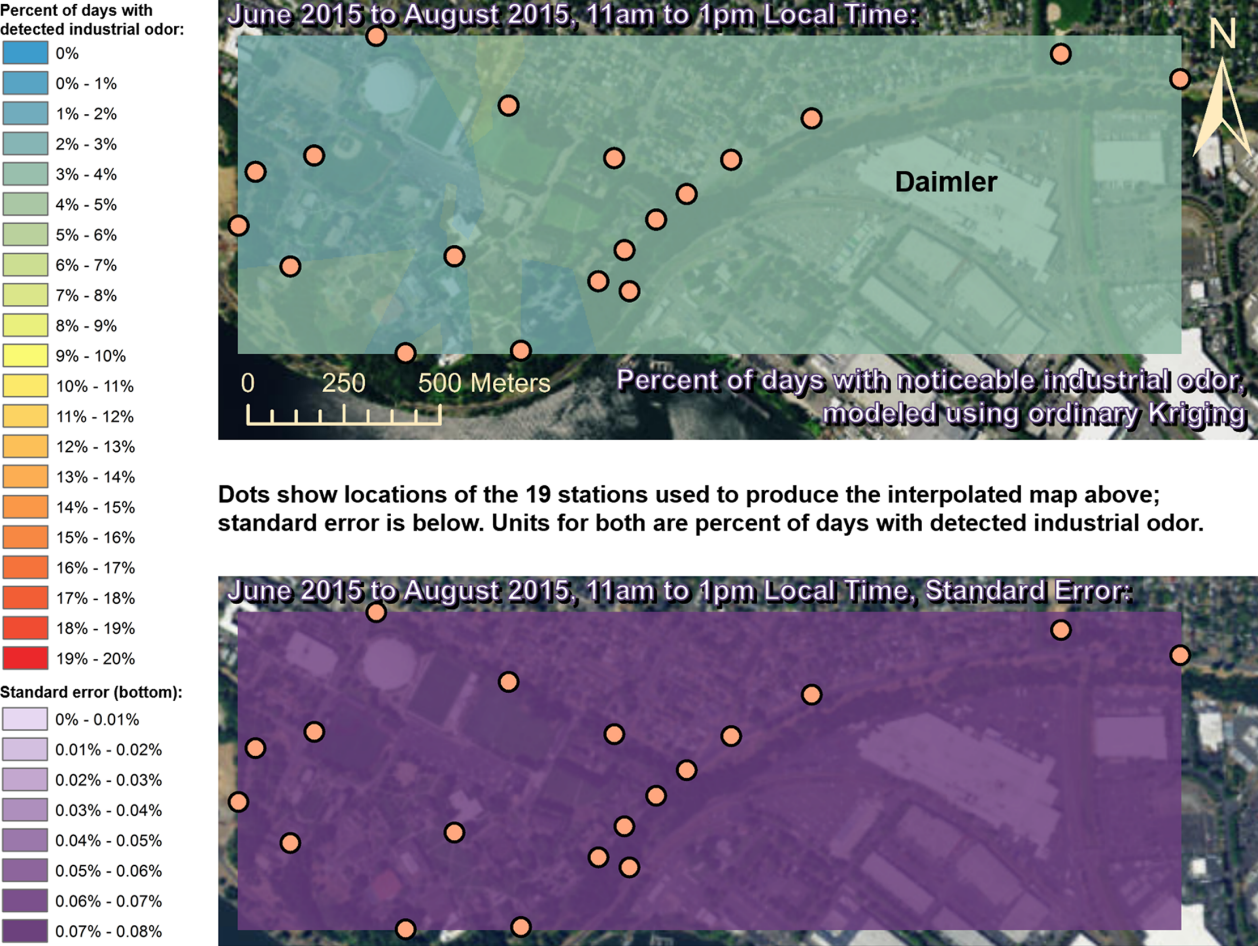
Frequencies of industrial odor detections (above) and standard error (below) during midday in summer. The spatial distribution of odor detections shows no clear pattern during this period. The bins here include all the values (no locations exceeded 20% of days with detected odors, or a standard error of 0.08%). Basemap from Esri’s ArcGIS.

**Fig 10:**
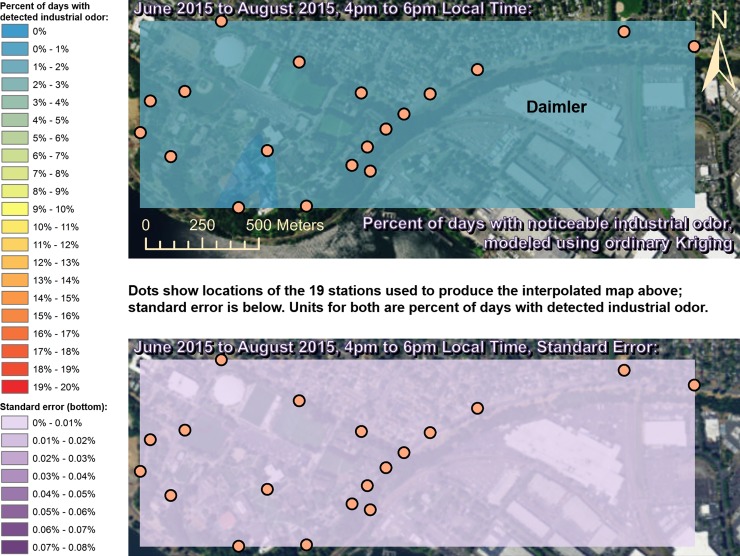
Frequencies of industrial odor detections (above) and standard error (below) during evening in summer. The spatial distribution of odor detections shows no clear pattern during this period. The bins here include all the values (no locations exceeded 20% of days with detected odors, or a standard error of 0.08%). Basemap from Esri’s ArcGIS.

**Fig 11:**
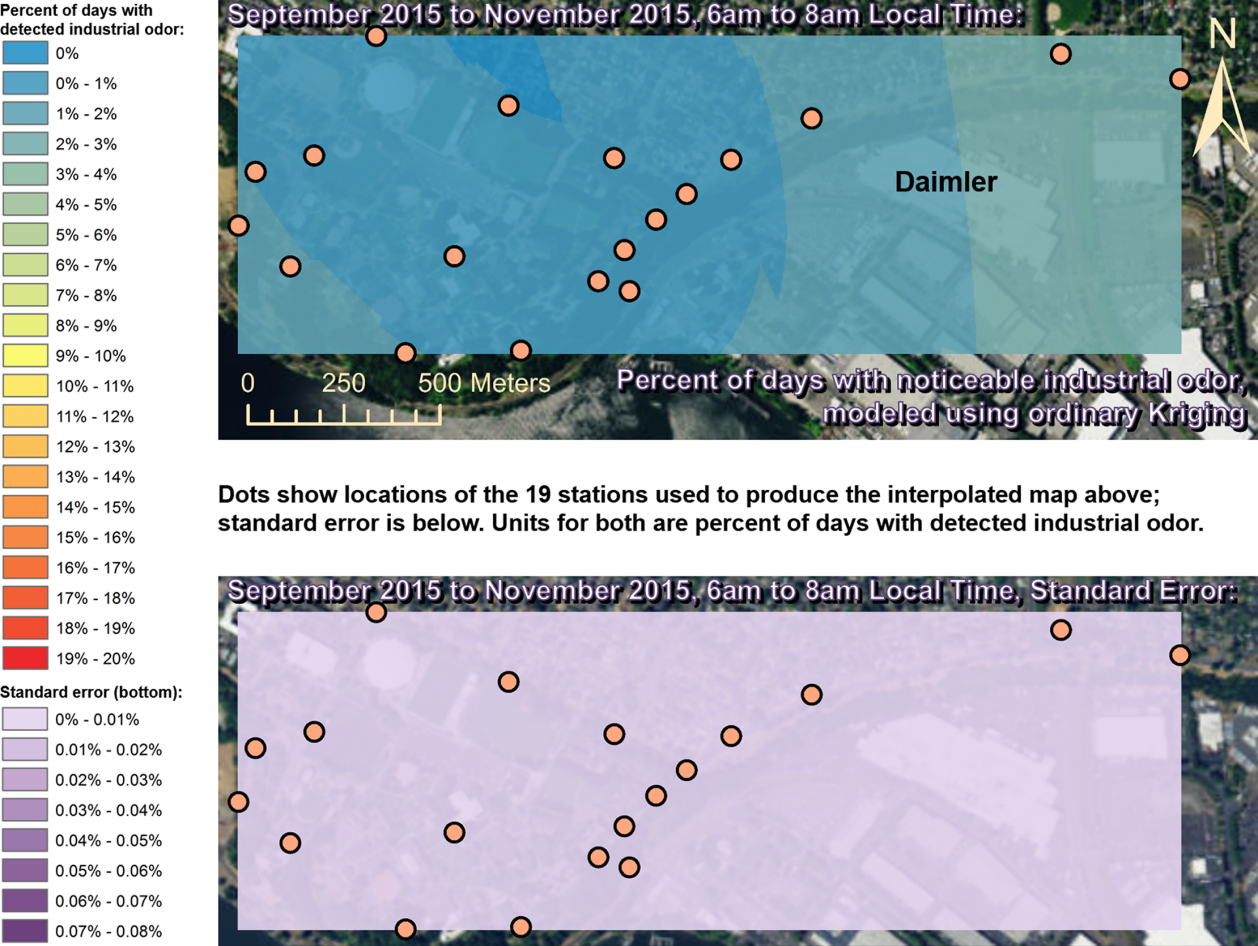
Frequencies of industrial odor detections (above) and standard error (below) during morning in fall. Odor detections generally decrease towards the northwest region of the map during this period. These frequencies of odor detections are all much larger than the standard errors from the interpolation shown here (below) indicating a high signal-to-noise ratio. As expected, standard errors generally increase with increasing distance from the stations used to produce the interpolation (the dots shown here). The bins here include all the values (no locations exceeded 20% of days with detected odors, or a standard error of 0.08%). Basemap from Esri’s ArcGIS.

**Fig 12:**
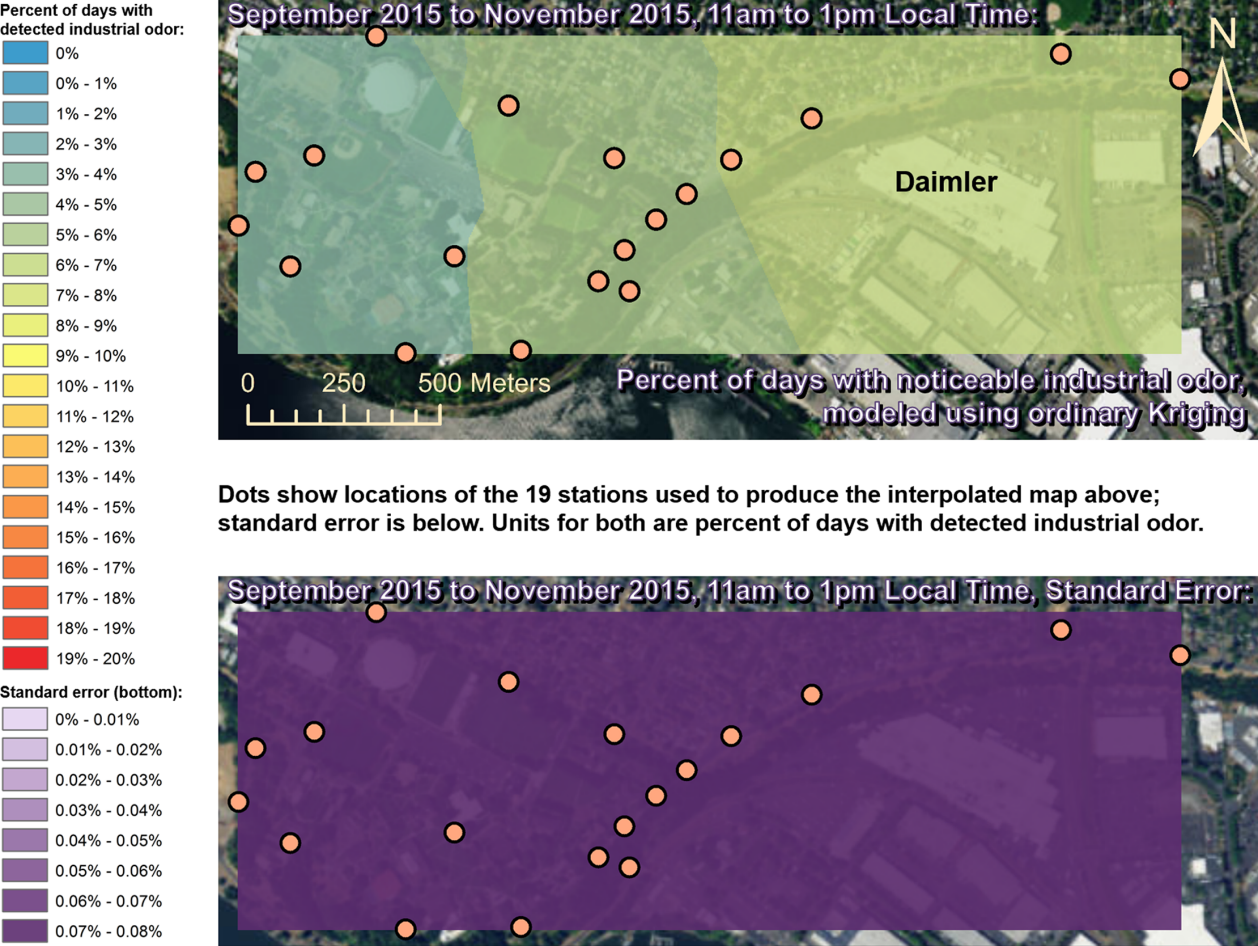
Frequencies of industrial odor detections (above) and standard error (below) during midday in fall. Odor detections generally decrease towards the western region of the map during this period. These frequencies of odor detections are all much larger than the standard errors from the interpolation shown here (below) indicating a high signal-to-noise ratio. As expected, standard errors generally increase with increasing distance from the stations used to produce the interpolation (the dots shown here). The bins here include all the values (no locations exceeded 20% of days with detected odors, or a standard error of 0.08%). Basemap from Esri’s ArcGIS.

**Fig 13:**
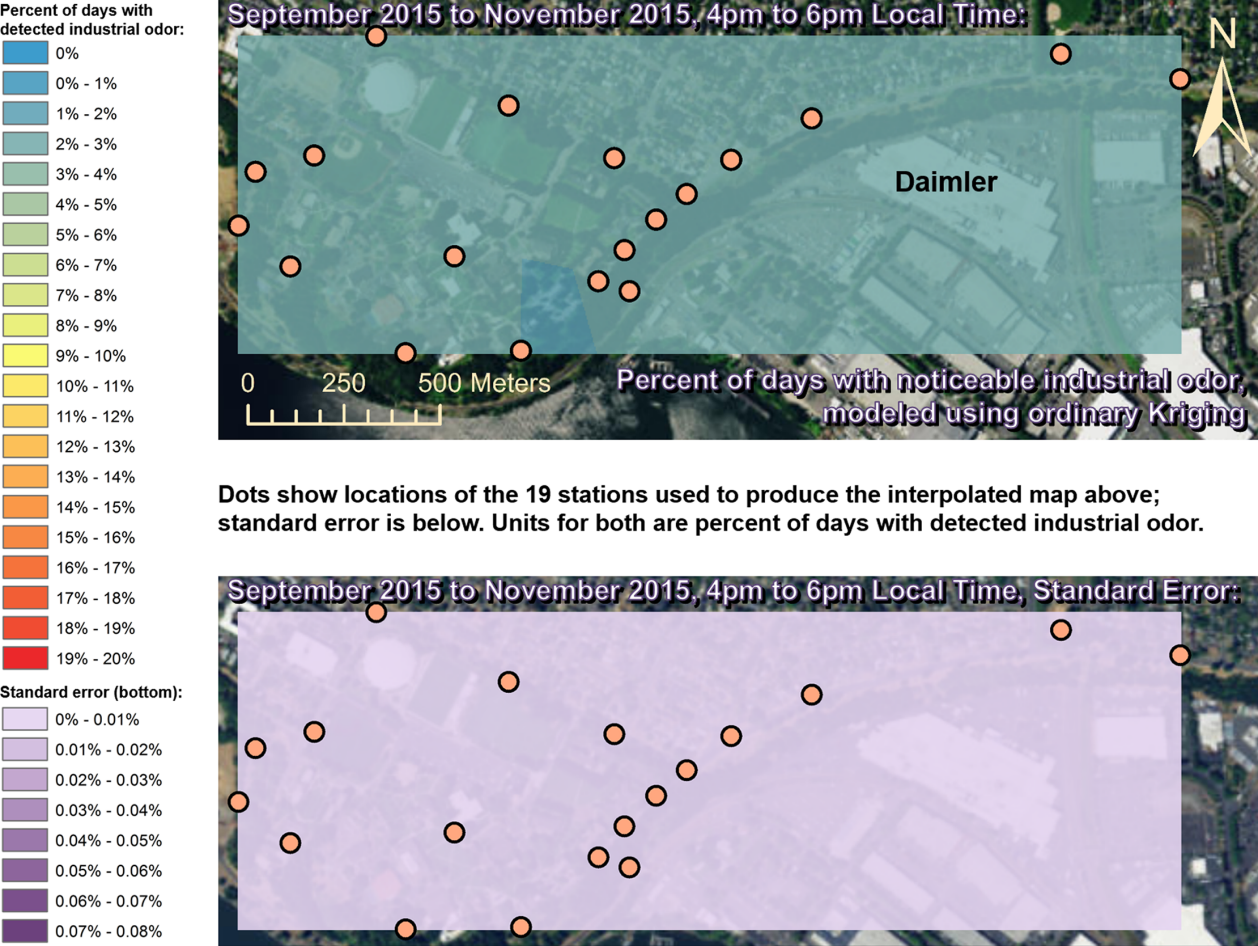
Frequencies of industrial odor detections (above) and standard error (below) during evening in fall. The spatial distribution of odor detections shows no clear pattern during this period. The bins here include all the values (no locations exceeded 20% of days with detected odors, or a standard error of 0.08%). Basemap from Esri’s ArcGIS.

Spatially interpolated maps of industrial odor frequencies are shown for all the seasons and times of day when this study made measurements in Figs 2–13, each with a corresponding standard error map. These standard errors are all below 0.08% of observations, making them much smaller than the frequencies of detected odors, which exceed 19% of observations in some times and locations (Fig 2). This suggests the broad spatial patterns modeled by Ordinary Kriging fit the input measurements very well. The areas northwest of Daimler experienced far more industrial odors during the morning, midday, and evening in winter (Figs 2–4), and morning and midday in spring (Figs 5 and 6), as compared to other times of day and seasons (Figs 7–13). This temporal variability highlights the importance of a sampling regime that includes multiple odor observations per day over the course of an entire year to assess the status of odors in the neighborhood.

**Fig 14:**
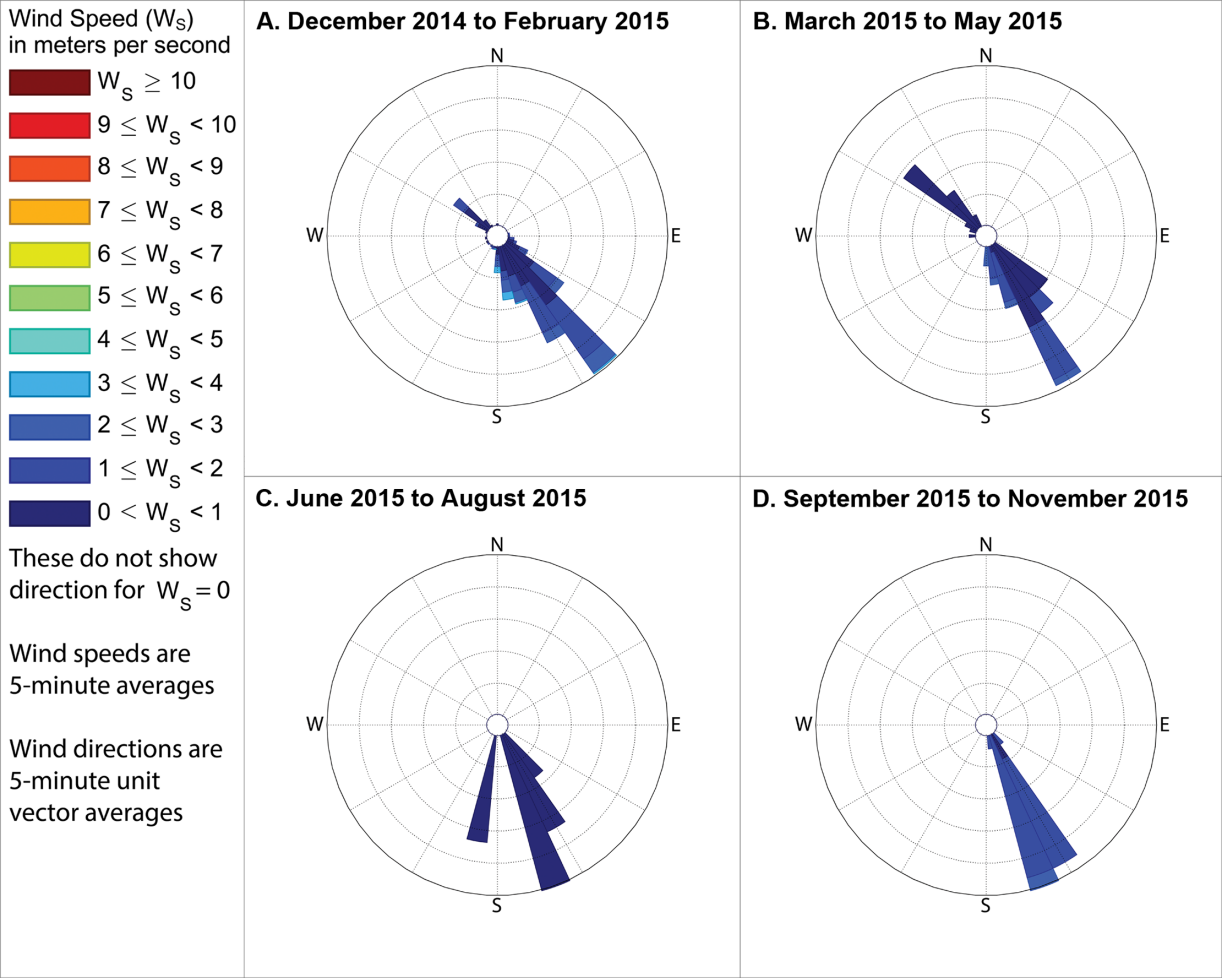
Winds measured between 6am and 8am local time when industrial odors were detected. These show directions from which the wind blew when industrial odors were detected at either station 13 or 14 during mornings in (A) winter, with the outermost ring indicating a 20% frequency, (B) spring, with the outermost ring representing a 10% frequency, (C) summer, with the outermost ring representing a 12% frequency, and (D) fall, with the outermost ring indicating a 24% frequency. These frequencies, and those stated in the text, include wind directions even when the measured wind speed was 0 m s^-1^, except where specifically indicated.

**Fig 15:**
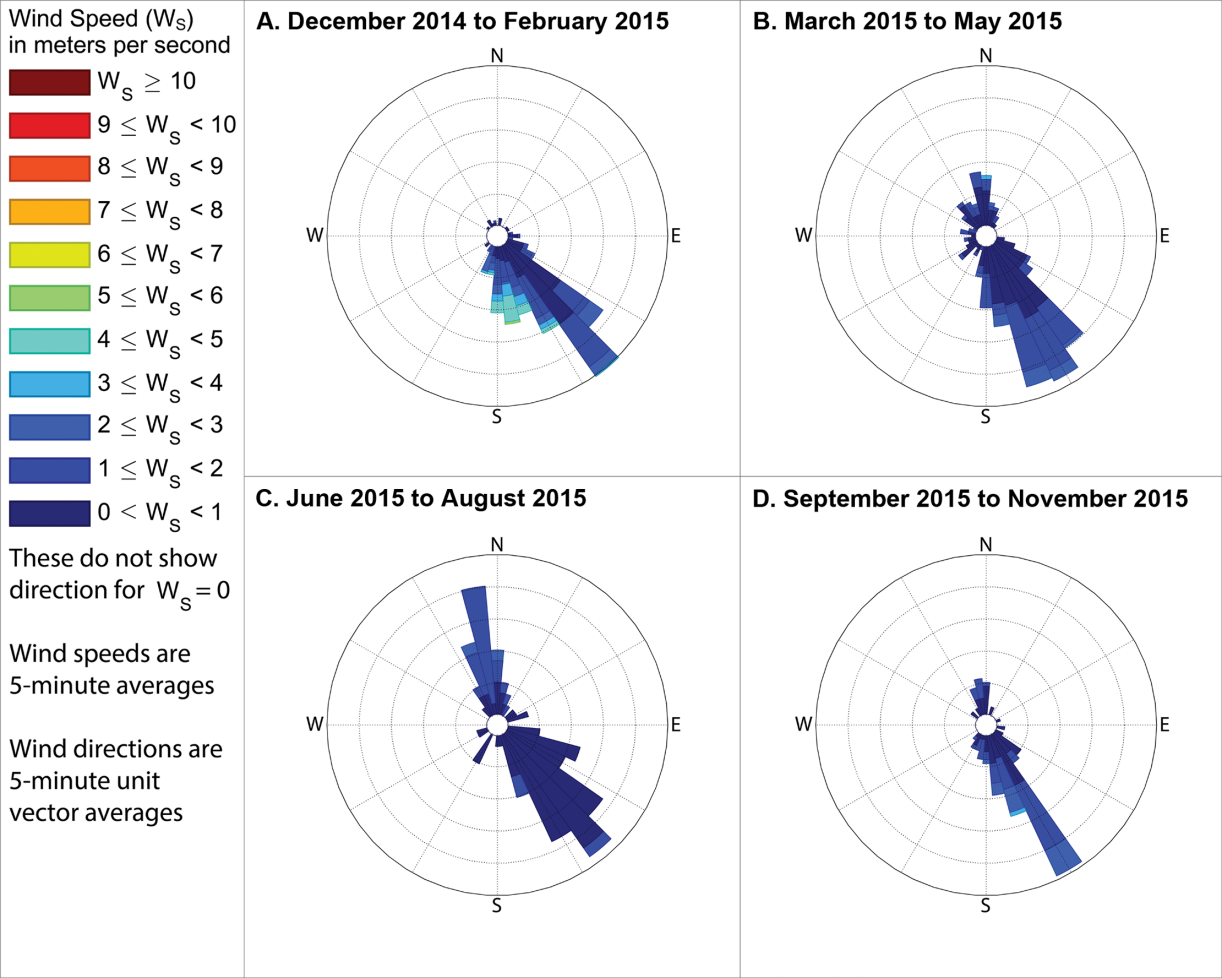
Winds measured between 11am and 1pm local time when industrial odors were detected. These show directions from which the wind blew when industrial odors were detected at either station 13 or 14 during midday in (A) winter, with the outermost ring indicating a 16% frequency, (B) spring, with the outermost ring representing an 11% frequency, (C) summer, with the outermost ring representing an 11% frequency, and (D) fall, with the outermost ring indicating a 20% frequency. These frequencies, and those stated in the text, include wind directions even when the measured wind speed was 0 m s^-1^, except where specifically indicated.

**Fig 16:**
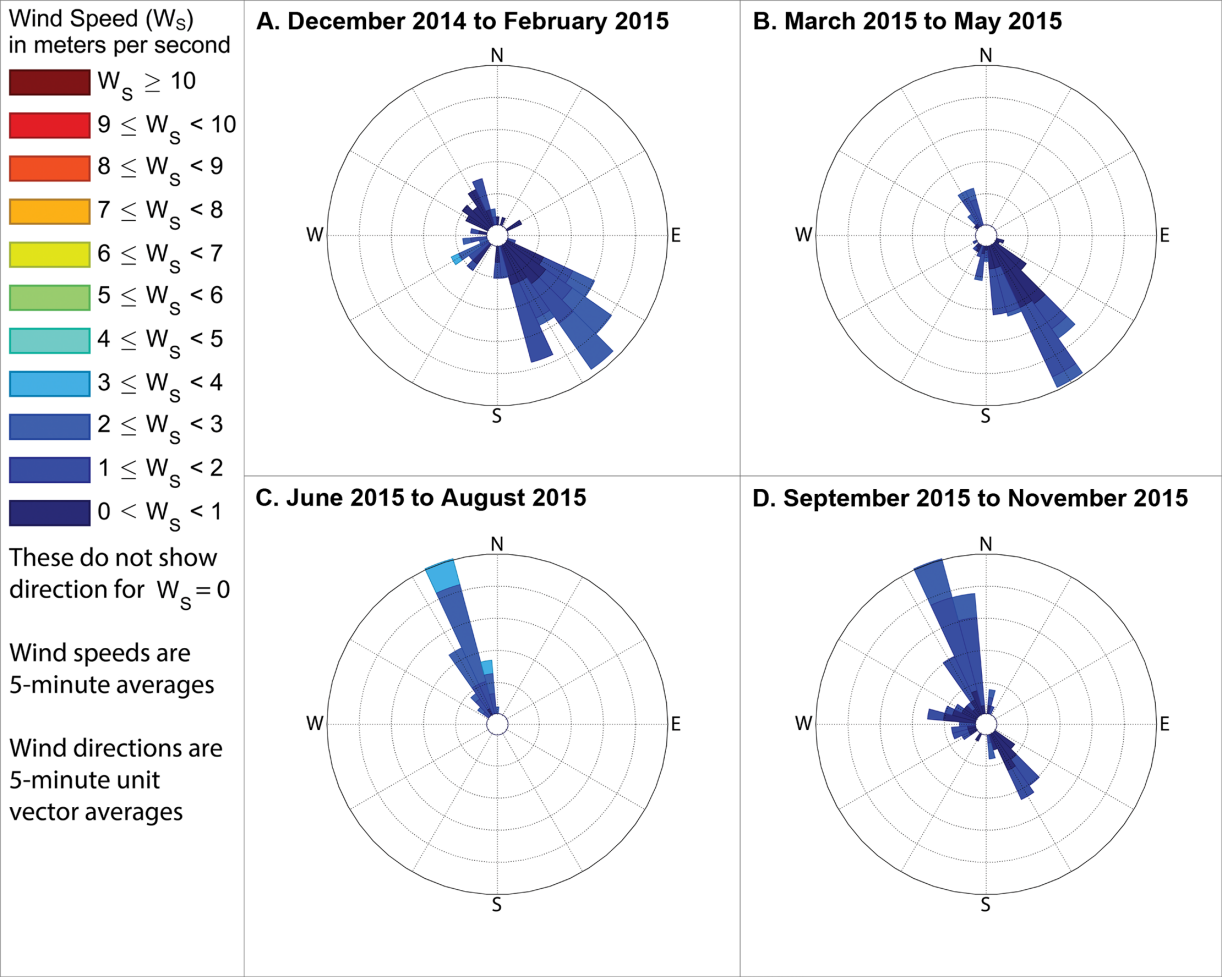
Wind directions from 4pm to 6pm local time when industrial odors were detected. These show directions from which the wind blew when industrial odors were detected at either station 13 or 14 during evenings in (A) winter, with the outermost ring indicating a 10% frequency, (B) spring, with the outermost ring representing a 23% frequency, (C) summer, with the outermost ring representing a 48% frequency, and (D) fall, with the outermost ring indicating a 16% frequency. These frequencies, and those stated in the text, include wind directions even when the measured wind speed was 0 m s^-1^, except where specifically indicated.

**Fig 17:**
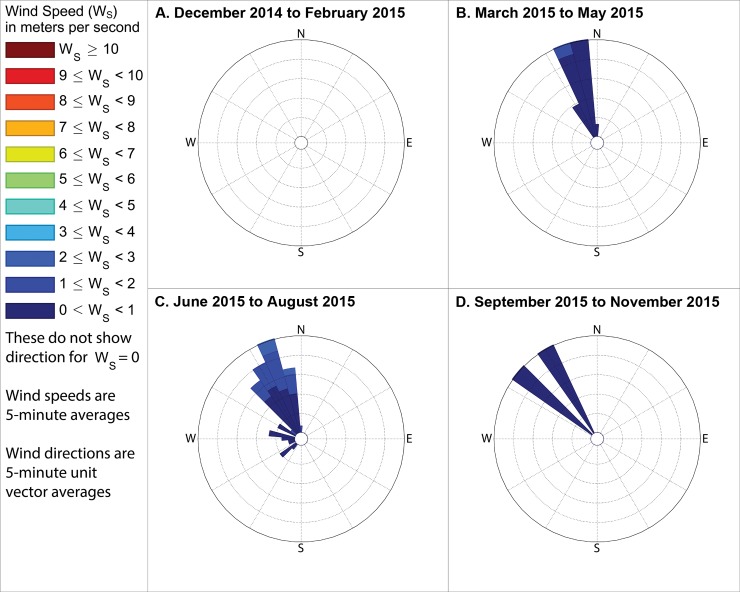
Winds measured between 6am and 8am local time when food preparation odors were detected. These show directions from which the wind blew when food preparation odors were detected at either station 13 or 14 during mornings in (A) winter, when no food preparation odors were detected, (B) spring, with the outermost ring representing a 32% frequency, (C) summer, with the outermost ring representing a 20% frequency, and (D) fall, with the outermost ring indicating a 4% frequency. These frequencies, and those stated in the text, include wind directions even when the measured wind speed was 0 m s^-1^, except where specifically indicated.

**Fig 18:**
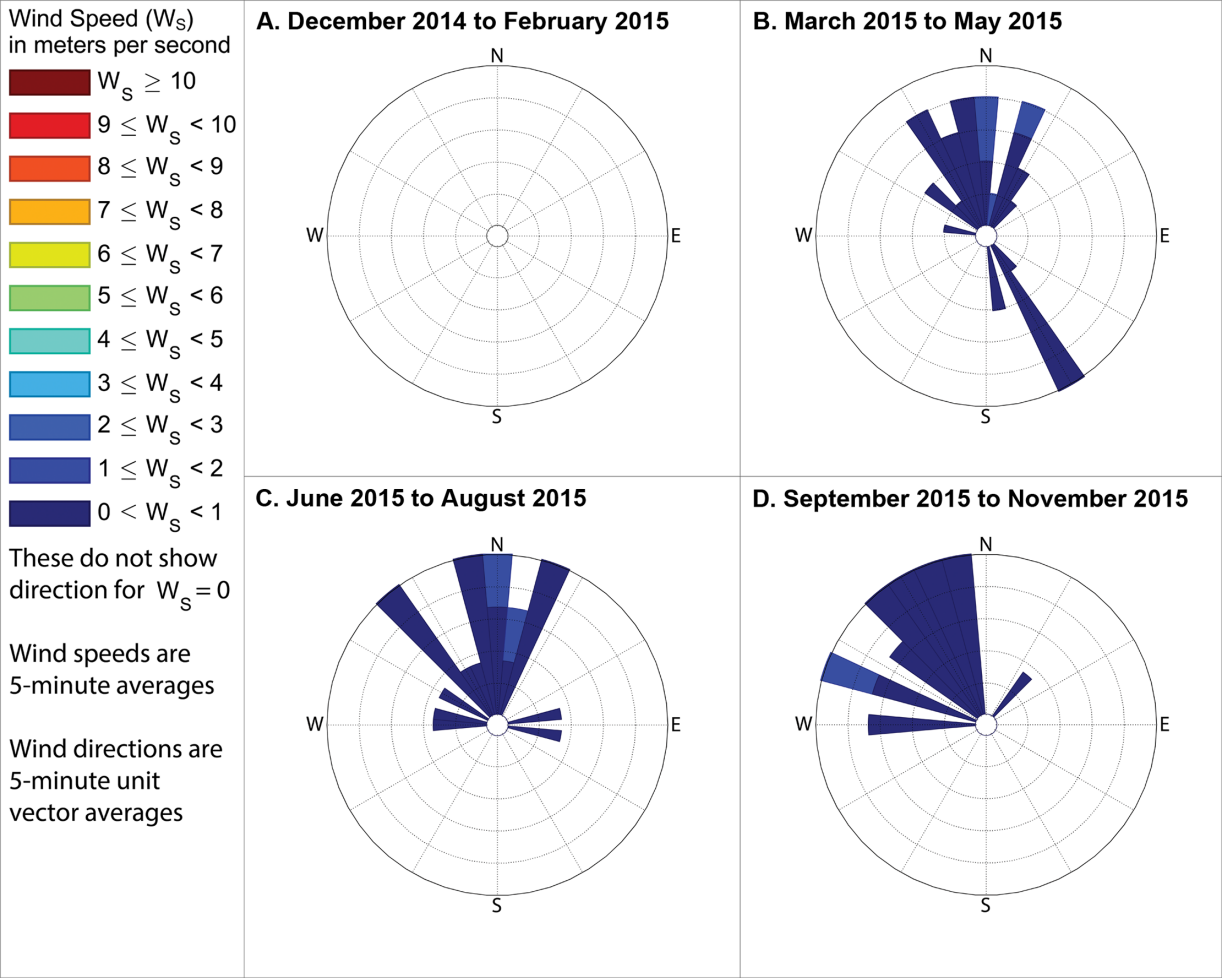
Winds measured between 11am and 1pm local time when food preparation odors were detected. These show directions from which the wind blew when food preparation odors were detected at either station 13 or 14 during midday in (A) winter, when no food preparation odors were detected, (B) spring, with the outermost ring representing a 10% frequency, (C) summer, with the outermost ring representing a 12% frequency, and (D) fall, with the outermost ring indicating a 12% frequency. These frequencies, and those stated in the text, include wind directions even when the measured wind speed was 0 m s^-1^, except where specifically indicated.

**Fig 19:**
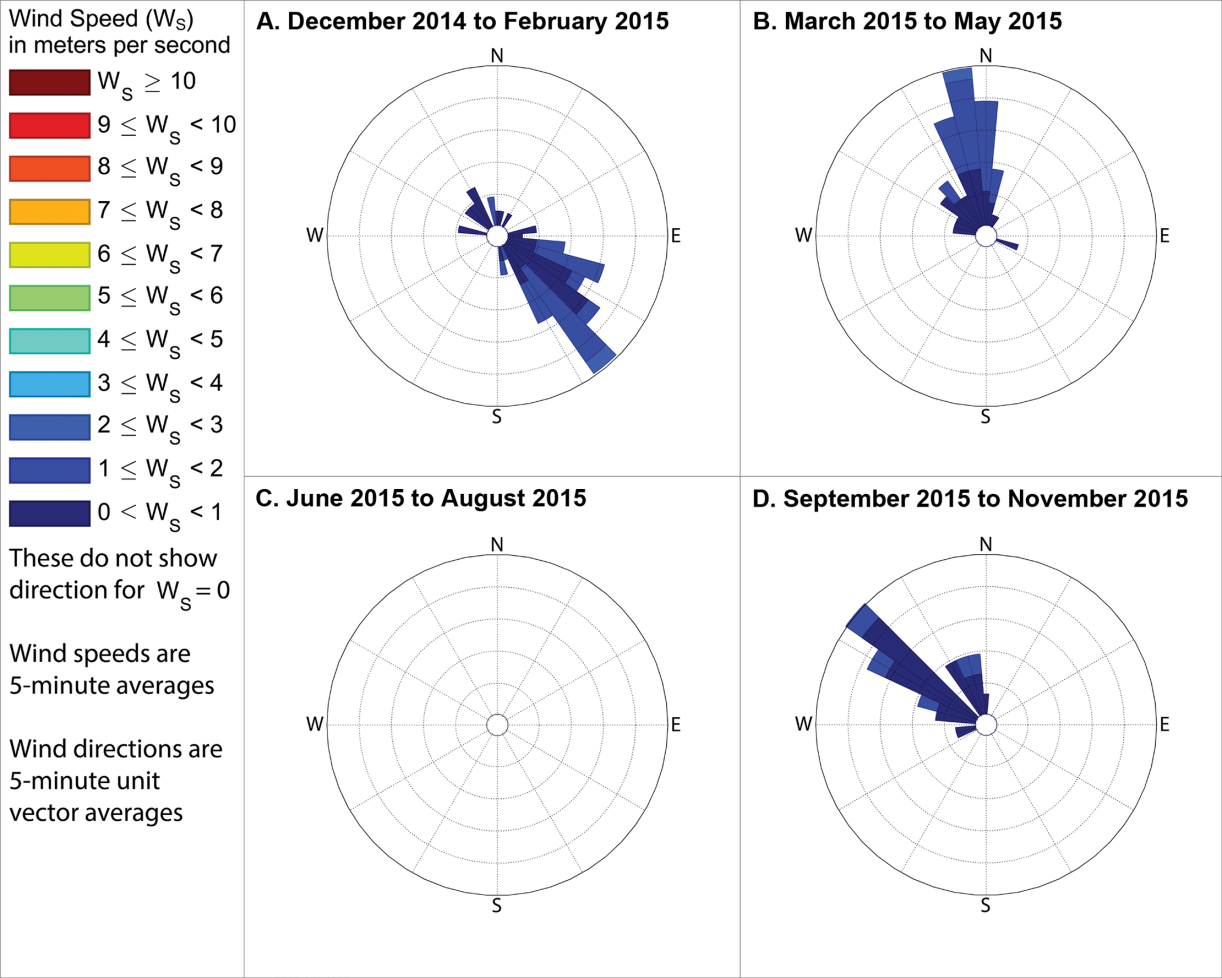
Winds measured between 4pm and 6pm local time when food preparation odors were detected. These show directions from which the wind blew when food preparation odors were detected at either station 13 or 14 during evenings in (A) winter, with the outermost ring indicating a 7% frequency, (B) spring, with the outermost ring representing a 19% frequency, (C) summer, when no food preparation odors were detected during the evening, and (D) fall, with the outermost ring indicating a 16% frequency. These frequencies, and those stated in the text, include wind directions even when the measured wind speed was 0 m s^-1^, except where specifically indicated.

Although odor frequencies vary substantially by season and time of day, by far the most industrial odor detections occurred at stations 13 and 14 (see station numbering in Fig 1) and therefore this study then analyzed the winds by season and time of day when these stations recorded industrial odors (Figs 14–16). To assess the reliability of the odor type classification, we also compared these to the winds when food preparation odors were recorded at stations 13 and 14. Winds were mostly from the southeast when industrial odors were recorded at these stations, supporting the hypothesis that the Daimler plant, which is immediately southeast of these stations, is the source of much of these industrial odors. Conversely, winds were mostly out of the northwest or northeast when food preparation odors were detected at these stations (Figs 17–19), suggesting that the odor categorizations are reliable because food preparation occurs frequently in the houses immediately northwest and northeast of these stations.

The following subsections describe these measured winds in detail, focusing on the percent of observations when winds were from the southeast during times when industrial odors were detected at stations 13 or 14 because Daimler is immediately southeast of these stations. We then assess reliability of odor categorization with subsections examining the percent of observations when winds were from the northwest or northeast during times when food preparation odors were detected at stations 13 or 14 (houses where food preparation occurs frequently are immediately northwest and northeast of those stations). Because the wind direction sensor used by this study may not provide accurate or representative directions in very low wind speeds, such as those below the minimum speed that our wind speed sensor can reliably measure (Table 1), we present frequencies for winds in two formats: first counting all directional measurements, and then in parenthesis counting only observations where the wind speed sensor recorded a speed above 0 m s^-1^. The following subsections organize these descriptions of winds by season, while Figs 14–19 group these results by time of day, to facilitate analyses on both of these temporal scales.

### Winds when industrial odors were detected at station 13 or 14

#### During winter

When industrial odors were detected at either station 13 or 14 during winter mornings, winds blew from the quadrant between due south and due east, hereinafter referred to as the southeast quadrant, 79.4% of the time (83.6% counting only observations with nonzero wind speed), as shown in Fig 14 A. When industrial odors were detected at either station 13 or 14 during midday (Fig 15A) winds blew from the southeast quadrant 72.2% of the time (83.2% counting only observations with nonzero wind speed). When industrial odors were detected during evening at either station 13 or 14 (Fig 16A) winds blew from the southeast quadrant 40.5% of the time (62.2% counting only observations with nonzero wind speed).

#### During spring

In the spring when industrial odors were detected at either station 13 or 14 during morning (Fig 14B), winds blew from the southeast quadrant 43.6% of the time (69.9% counting only observations with nonzero wind speed). In the spring when industrial odors were detected at either station 13 or 14 during midday (Fig 15B), winds blew from the southeast quadrant 59.4% of the time (61.2% counting only observations with nonzero wind speed). In the spring when industrial odors were detected at either stations 13 or 14 in the evening (Fig 16B), winds blew from the southeast quadrant 74.8% of the time (73.6% counting only observations with nonzero wind speed).

#### During summer

In the summer when industrial odors were detected at either station 13 or 14 during morning (Fig 14 C) winds blew from the southeast quadrant 88% of the time (75% counting only observations with nonzero wind speed). In the summer when industrial odors were detected at either station 13 or 14 during midday (Fig 15C), winds blew from the southeast quadrant 56.8% of the time (56.7% counting only observations with nonzero wind speed). In the summer when industrial odors were detected at either stations 13 or 14 in the evening (Fig 16C), winds blew from the southeast quadrant 0% of the time (0% counting only observations with nonzero wind speed), though very few industrial odors were detected anywhere in the study area during summer evenings (Fig 10).

#### During fall

In the fall when industrial odors were detected at either stations 13 or 14 during morning (Fig 14D), winds blew from the southeast quadrant 50% of the time (100% counting only observations with nonzero wind speed). In the fall when industrial odors were detected at either stations 13 or 14 in the midday (Fig 15D), winds blew from the southeast quadrant 62.4% of the time (67% counting only observations with nonzero wind speed). In the fall when industrial odors were detected at either stations 13 or 14 in the evening (Fig 16D), winds blew from the southeast quadrant 32.8% of the time (25% counting only observations with nonzero wind speed), though similar to the case with summer evenings, very few industrial odors were detected anywhere in the study area during fall evenings (Fig 13).

### Winds when food preparation odors were detected at station 13 or 14

#### During winter

In winter, no food preparation odors were detected at either stations 13 or 14 during the morning (Fig 17A) or midday periods (Fig 18A). When food preparation odors were detected at either stations 13 or 14 during evening (Fig 19A), winds blew from the quadrant between north and west, hereinafter referred to as the northwest quadrant, 44% of the time (17.7% counting only observations with nonzero wind speed) and from the quadrant between due north and due east, hereinafter referred to as the northeast quadrant, 9.6% of the time (8% counting only observations with nonzero wind speed).

#### During spring

In the spring when food preparation odors were detected at either stations 13 or 14 in the morning (Fig 17B), winds blew from the northwest quadrant 96% of the time (95% counting only observations with nonzero wind speed) and blew from the northeast quadrant 5% of the time (5% counting only observations with nonzero wind speed). In the spring when food preparation odors were detected at either stations 13 or 14 during midday (Fig 18B), winds blew from the northwest quadrant 36% of the time (48.5% counting only observations with nonzero wind speed) and blew from the northeast quadrant 54.2% of the time (28.5% counting only observations with nonzero wind speed). In the spring when food preparation odors were detected at either stations 13 or 14 during evening (Fig 19B), winds blew from the northwest quadrant 72% of the time (79% counting only observations with nonzero wind speed) and blew from the northeast quadrant 12.9% of the time (12.9% counting only observations with nonzero wind speed).

#### During summer

In the summer when food preparation odors were detected in the morning at either stations 13 or 14 (Fig 17B), winds blew from the northwest quadrant 85.3% of the time (87.5% counting only observations with nonzero wind speed) and blew from the northeast quadrant 0% of the time (0% counting only observations with nonzero wind speed). In the summer when food preparation odors were detected at either stations 13 or 14 during midday (Fig 18B), winds blew from the northwest quadrant 52% of the time (57.1% counting only observations with nonzero wind speed) and blew from the northeast quadrant 42.8% of the time (38% counting only observations with nonzero wind speed). No food preparation odors were detected at either stations 13 or 14 during summer evenings.

#### During fall

In the fall when food preparation odors were detected at either stations 13 or 14 in the morning (Fig 17D), winds blew from the northwest quadrant 12% of the time (100% counting only observations with nonzero wind speed) and blew from the northeast quadrant 50% of the time (0% counting only observations with nonzero wind speed). When food preparation odors were detected during midday at either stations 13 or 14 (Fig 18D), winds blew from the northwest quadrant 92% of the time (90% counting only observations with nonzero wind speed) and blew from the northeast quadrant 5% of the time (5% counting only observations with nonzero wind speed). When food preparation odors were detected at either stations 13 or 14 during evening (Fig 19D), winds blew from the northwest quadrant 80% of the time (90.3% counting only observations with nonzero wind speed) and blew from the northeast quadrant 25.8% of the time (3.2% counting only observations with nonzero wind speed).

### Uncertainty

While some outliers, such as those noted above, are present in the odor and wind datasets, the combination of all the analyses presented here still supports the hypothesis that Daimler is the main source of industrial odors in the study area. The uncertainties from the standard error maps in Figs 2–13 are very small relative to the patterns discussed here, but the uncertainty in the odor data is difficult to quantify. Table 2 shows that most of the odors detected by this study were not categorized as industrial, and thus if odors were consistently placed into the wrong categories, the effects of this on the results presented here could be substantial. However, the quantified uncertainty for the wind data in Table 1 is very small relative to the patterns discussed here, and the wind data therefore suggest the odor categorizations were reliable in that they fit well with the locations of Daimler producing paint odors while nearby houses produced food preparation odors. Moreover there is no reason to believe that odors as distinct to human olfaction as food versus paint/industrial odors were likely to be confused with one another by trained and experienced observers. Even considering all the sources of uncertainty described above, combining all the wind and odor data presented here still strongly suggests that Daimler’s facility, which paints trucks, is the source of much of the paint odor observed at stations 13 and 14 when the winds we measured would be pushing odors from Daimler’s facility towards those stations.

## Discussion

As already noted, our study provided the opportunity to attempt to disprove the hypothesis that Daimler is the main source of industrial odors detected in the nearby residential neighborhood by collecting data on odor type for a year, measuring winds throughout the year, and analyzing patterns in odors through Kriging along with their relationships to winds. Three results would allow that hypothesis to be considered disproven: (1) Kriging might indicate industrial odors are not statistically more likely to be present near Daimler than in other parts of the study area, (2) the type of odors more frequently observed near Daimler may be inconsistent with Daimler being their source (*e*.*g*. are they paint odors, or from a process not present at Daimler?), or (3) winds might not be consistent with odors being carried from the Daimler facility to the residential neighborhood. The hypothesis that Daimler is the main source of industrial odors in the neighborhood survived all three of these tests.

This study’s findings differ from those of the earlier study by Oregon’s DEQ [1]. Where DEQ had more limited resources and hence less data, we were able to collect data on odors three times per day for a year, at 19 locations, and local wind direction and speed data continuously. Our study analyzed 124,785 data points (19,665 odor observations plus 105,120 wind measurements), while the DEQ study included only 760 data points collected intermittently, and did not account for spatial or temporal biases in their analysis. The DEQ study’s sampling methods created inadvertent diurnal and seasonal biases because they conducted far more observations at some times of year versus others and did not account for this in their analysis [1]. For example, the DEQ study observed for over 1,100 minutes in June, but under 500 minutes in December, which underestimates true odor frequency, because as our study shows, industrial odors in this area occur far more often in winter months than they do in summer. Similarly, the DEQ over-sampled during some times of day and under-sampled during others, thus producing a time-of-day bias. Conversely, our study made the same number of observations at all times of day that we investigated. Finally, the DEQ study does not correct for its spatial bias where sampling locations are not distributed evenly throughout space across the study area. Our study also did not sample all locations or distribute sampling stations evenly because of obstacles in the way such as steep terrain and private properties, but our study accounted for this through the spatial interpolation in Figs 2–13. While Daimler is not the only source of odors detected in the study area, our analyses do not reveal any other major source of industrial odors with such a strong corroboration from spatial statistics, odor type, and winds across multiple times of day and seasons.

The DEQ study concluded that Daimler’s facility was not causing a nuisance order, but further analysis suggests that the data available were insufficient to support that conclusion. The DEQ study divided their 24 odor detections by their 760 observations (yielding 3.2%) and used this number to reach its conclusion, though that 3.2% does not include any spatial or temporal information. Such a simple calculation could be artificially biased lower by scheduling more observations at times of day or times of the year when odors were less likely to be present, or at locations where odors were less likely to be present. According to our full spatial analyses by time of day and season, this was indeed a fundamental flaw in the DEQ study’s analysis: most of DEQ’s observations occurred at times and locations where odors were less likely to be present, while DEQ provided no corrections for uneven spatiotemporal sampling. The DEQ study also states in reaching its conclusion that DEQ staff spent 10,213 minutes observing for odors, and detected an odor for 187 of those minutes (1.8% of the time they spent observing) [1]. Olfactory fatigue, inconsistencies in sampling durations, locations, and timing, plus small sample sizes, could explain why this number (1.8%) is so different from their other calculation of 3.2%. The DEQ study makes no assessment of uncertainty, though substantial uncertainty clearly exists in their findings. However, the DEQ study states its conclusion in no uncertain terms as follows: “DEQ’s Northwest Region concludes that the evidence gathered through its October 2014 through October 2015 nuisance odor investigation of Daimler Trucks North America, L.L.C.’s North Portland facility is sufficient to document that during the time of DEQ’s investigation, the facility was not causing a nuisance odor.” Our analyses suggest that the DEQ’s conclusion is not supported by the available data.

Many scientific studies of odors elsewhere have evaluated or employed the same methods used by our study, or similar methods, and found them to be reliable. These studies range from sniffing team campaigns around landfill sites [33], to olfactometry for odor determination at wastewater collection systems [34], and trained resident observers near swine farms [35]. Some studies have used field-sampled measurements of odors like the ones we made to validate mathematical dispersion models like CALPUFF and ISC3/ISCST3 at places such as beef cattle feedlots [36] and commercial pig units [37], and typically find good agreement between models and field measurements [38]. New odor models continue to appear [39], and existing odor models are frequently refined [40], so better procedures and more datasets from gathering/analyzing odor data such as through studies like ours should lead to improved validation and thus better models. Odor measurements following VDI 3940 methods have also been statistically analyzed with Kriging to assess the spatial extent of odor nuisance [17], and our study employs Kriging for statistical analysis as well, but provides information about temporal variability in Kriging results (Figs 2–13) that is not presented by some other studies [18]. Likewise, very few studies present analyses of odor and wind by time of day across multiple seasons to the extent that our study does (Figs 2–19) [9], in some cases because those studies did not gather odor data across a full year as ours did.

Our case study demonstrates the importance of using a full year of odor data collection, and other lessons that investigators in other locations could apply to their odor studies. Some studies elsewhere have used shorter collection periods such as six months [18], typically including cold and warm seasons about equally [15], but for climates similar to Portland’s, this could produce a seasonal bias because the odor distributions we observed in spring (Figs 5–7) and fall (Figs 11–13) are different, both on a daily basis and by time of day. This is not surprising because the winds we measured in spring were different from the winds we measured in fall (Figs 14–19), and fits with other studies of our region’s climatic variability in winds [2] and other atmospheric parameters [3] on seasonal and diurnal timescales. However, the higher costs associated with a year-long monitoring period have been pointed out by others [9], and may not be worthwhile in climates with less variability than the large differences in odor occurrence by season that we documented in Portland. As is the case with most odor studies involving field sampling [9], our study and DEQ’s are both limited in that they only covered a single year, and thus do not incorporate year-to-year variability in atmospheric conditions. To fully understand odors here, we would need to perform a multi-year study, though resource limitations prevent a multi-year study at this time.

The patterns in season and time of day shown in Figs 2–13 provide potential solutions to some of the odor problems in the area: Daimler production occurs year-round from morning to evening most days, yet atmospheric conditions appear not to cause Daimler’s emissions to reach high concentrations during the summer, so this would be the best time of year for production to occur without subjecting nearby residents to frequent odors. Similarly, some combinations of season and time of day appear to have atmospheric conditions that concentrate Daimler’s emissions in the University Park neighborhood, and our study therefore recommends that emission-causing production be effectively filtered, limited, or eliminated during those times. Even during the time of year with the most frequent industrial odors (winter mornings) we detected industrial odors just under 20% of the time near Daimler, so there is potential for this study’s spatiotemporal analyses, perhaps combined with accurate wind forecasts, to provide a solution to this problem by suggesting times when odor-producing operations would not impact nearby residents. Daimler could then continue production at least 80% of the time as-is, and reschedule odor-producing operations away from other times when atmospheric conditions would cause emissions to create odor problems in the neighborhood.

It is unfortunate that few scientific studies of odors present measurements of odors and winds across multiple times of day in all seasons to the extent we do in Figs 2–16 [9], because our analyses of odors by time of day and season allowed us to identify this potential for a simple rescheduling of operations to solve the neighborhood’s odor problems. Our findings thus suggest that other scientific studies of odors should include similarly thorough temporal coverage and analyses, as these could reveal solutions to odor problems in some other areas as well. Environmental odors pose challenges in many nations [41] and can cause health problems [42], so scientific studies of environmental odors are important [43]. Thus our study has the potential for broad contributions to future scientific odor surveys and potentially science-driven odor policies [44] in many regions worldwide [45] by demonstrating the importance of thorough temporal coverage and analysis in areas with significant seasonal variability.

## Conclusions

Our results support the hypothesis that much of the industrial odor noted by residents in University Park comes from the Daimler Trucks North America LLC manufacturing facility. Winds during industrial odor events blew from the southeast a majority of the time during most seasons and times of day (Figs 14–16) suggesting that winds blew over the Daimler plant and carried the plant’s emissions into University Park, where they built to high enough concentrations to produce detectable odors. Our results differ from those of Oregon’s DEQ, which concluded that the Daimler plant was not causing a nuisance odor [1]. Our results also suggest a possible solution to this problem by demonstrating which combinations of season and time of day rarely cause odors to reach high concentrations in the University Park neighborhood, and thus providing valuable information that could help in rescheduling odor-producing operations at the Daimler plant to occur only at those times.

The authors of this study hope our analyses help Daimler and other stakeholders in the area, such as the residents of University Park, to reach a mutually agreeable solution such as this, but the winds and odor analyses we present here strongly suggest that much of the industrial odors in the area come from Daimler. Finally, this study has created a framework for: 1) identifying sources of industrial odors, 2) analyzing spatial and temporal patterns in the presence/absence of industrial odors, and 3) assessing uncertainty in the spatial interpolation and qualitative categorization of odors in these analyses. We believe that the framework developed by this study can be applied to help other areas move towards solving their industrial odor problems as well.

## Supporting information

S1 Dataset Wind data measured by this study. (XLSX)

S2 Dataset Zip file with odor data measured by this study. (ZIP)
